# Longitudinal omics data analysis: approaches and applications

**DOI:** 10.1016/j.csbj.2026.01.001

**Published:** 2026-01-05

**Authors:** Ali Reza Taheriyoun, Allen Ross, Abolfazl Safikhani, Damoon Soudbakhsh, Ali Rahnavard

**Affiliations:** a Department of Biostatistics and Bioinformatics, The George Washington University, Washington, DC 20052, USA; b Department of Statistics, George Mason University, Fairfax, VA 22030, USA; c Department of Mechanical Engineering, Temple University, Philadelphia, PA 19122, USA

**Keywords:** Balanced design, Differential expression analysis, Longitudinal omics data, Mixed effect model, Nonparametric estimation, Temporal dynamics, Time-course data

## Abstract

Longitudinal omics data (LOD) analysis is essential for understanding the dynamics of biological processes and disease progression over time. This review explores various statistical and computational approaches for analyzing such data, emphasizing their applications and limitations. The main characteristics of longitudinal data, such as imbalance, high-dimensionality, and non-Gaussianity are discussed for modeling and hypothesis testing. We discuss the properties of linear mixed models (LMM) and generalized linear mixed models (GLMM) as foundational tools in LOD analyses and highlight their extensions to handle the obstacles in the frequentist and Bayesian frameworks. We differentiate dynamic data analysis between time-course and longitudinal analyses, covering functional data analysis (FDA) and replication constraints. We explore classification techniques, single-cell studies as exemplary omics longitudinal studies, survival modeling, and multivariate methods for clinical/biomarker-based applications. Emerging topics, including data integration, clustering, and network-based modeling, are also discussed. We categorize the state-of-the-art approaches applicable to omics data, highlighting how they address the data features. This review serves as a guideline for researchers seeking robust strategies to analyze LOD effectively, which is usually complex.

## Graphical Abstract

**Figure:**
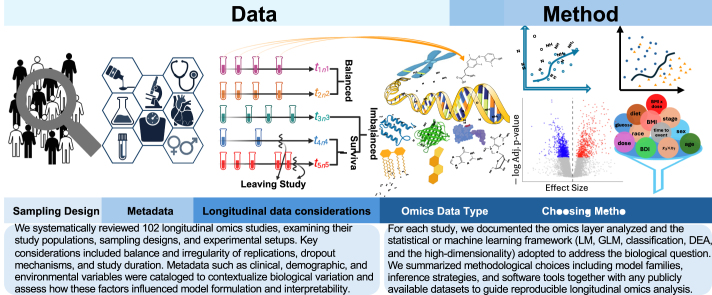

## 1 Introduction

**Fig. 1:**
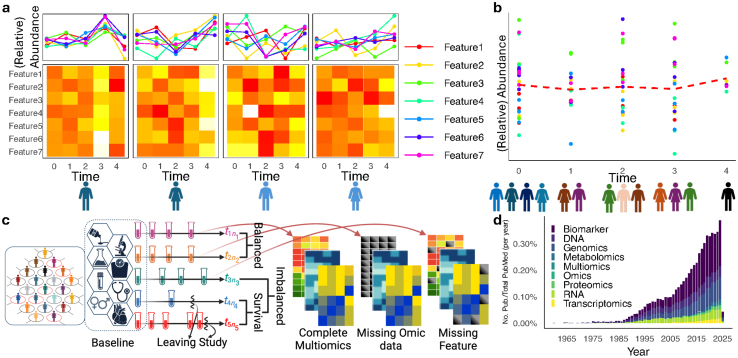
Overall study designs and omics measurements. **a** shows the basic structure of longitudinal data with repeated measurements per subject. In contrast, **b** shows a time-course study where ‘time’ is a covariate without repeated measurements. **c** illustrates multiomics studies with imbalanced sampling designs and missing data. The first two subjects are balanced despite irregular time points, while subjects 4 and 5 drop out, leading to imbalance. **d** shows the trend of publications based on longitudinal omics studies within the last 8 decades in PubMed databases generated by pubSight.

Longitudinal data consist of repeated measurements from multiple subjects over time. Unlike time-course data, which track a realization of a stochastic process, longitudinal data are sparse and subject-dependent (Fig. 1**a** and **b**). We explicitly distinguish between time-course and longitudinal study designs throughout this review, as formally defined and illustrated in Section 2.6. Biomarker interactions can enhance detection power [1], such as correlated metabolites in diabetes studies. However, full multivariate models for serial measurements introduce high-dimensional estimation challenges. A practical alternative for univariate outcomes is incorporating random effects into fixed effect models, such as the linear mixed model [2] (LMM).

Despite challenges, longitudinal studies provide robust statistical tests, reducing noise compared to independent samples [3], with lower variation in the estimates [4] and provide the progress path in treatment regimens. These have increased the demands for longitudinal omics data (LOD) in public health and pharmaceutical studies (Fig. 1**d**) with a sharp slope increase.

While many statistical and computational methods developed for LOD are motivated by technical considerations, their ultimate value lies in the biological and public health insights they enable. This has been reflected in the introductory chapters of most longitudinal data analysis texts [5], [6]. Compared with cross-sectional omics studies, longitudinal designs allow researchers to characterize dynamic biological processes, distinguish transient from persistent molecular changes, and infer temporal ordering between molecular events and phenotypic outcomes.

Specifically, longitudinal omics methods enable the identification of subject-specific molecular trajectories [7], [8], revealing heterogeneity in disease progression [9], [10], treatment response [11], [12], or recovery processes. Time-resolved analyses can uncover early molecular predictors of clinical outcomes, detect delayed or cumulative effects of exposures or interventions, and disentangle within-subject changes from between-subject variability.

In multiomics settings, longitudinal modeling further facilitates the investigation of coordinated temporal patterns across molecular layers, such as transcriptomic changes preceding metabolomic alterations or immune signatures evolving alongside clinical phenotypes. These dynamic relationships are often inaccessible to cross-sectional analyses, which conflate temporal variation with inter-individual differences [13]. Overall, LOD provide a framework for moving from static associations toward a mechanistic understanding of biological systems as they evolve over time.

This review examines analysis methods for longitudinal omics studies, highlighting challenges in addressing key scientific questions. We summarize research questions, available datasets, methodologies, and computational tools. Studies tackling multiple issues are categorized and discussed accordingly, with emphasis on their application to LOD. The provided file in the supplementary material (see also Section B of the supplementary materials for more information) categorizes studies for current methods and applications, and supplementary Figures S1 and S2 provide summaries of novel approaches and applications. As highlighted in Fig. 1**c** and described below, LOD analyses present challenges beyond the classic LMM approaches:

**Imbalanced or missing:** In genomics, imbalances arise from missing data, mostly irregular time points, or feature redundancy. Multiomics studies further complicate imbalances, as different omics layers may be missing. While most classic tools drop rows with missing values, some (e.g., JointAI [14] and bild [15]) offer alternatives, though model specification remains critical.

**Correlated outcomes:** Omics studies exhibit intra-subject and inter-feature correlations. While standard univariate models ignore the latter, multivariate methods and random effects can account for these correlations.

**Time-varying covariates:** Clinical and demographic factors change over time, influencing variance and effect estimation. Ignoring such changes can distort statistical inference.

**Nonlinearity:** Biological systems are rarely linear, and while linear models are commonly used, they may fail to capture complex biological feedback.

**Leave-out:** Subject dropout in clinical studies, due to treatment changes, study completion, or mortality, introduces biases, especially if dependent on omics measurements.

**Fig. 2:**
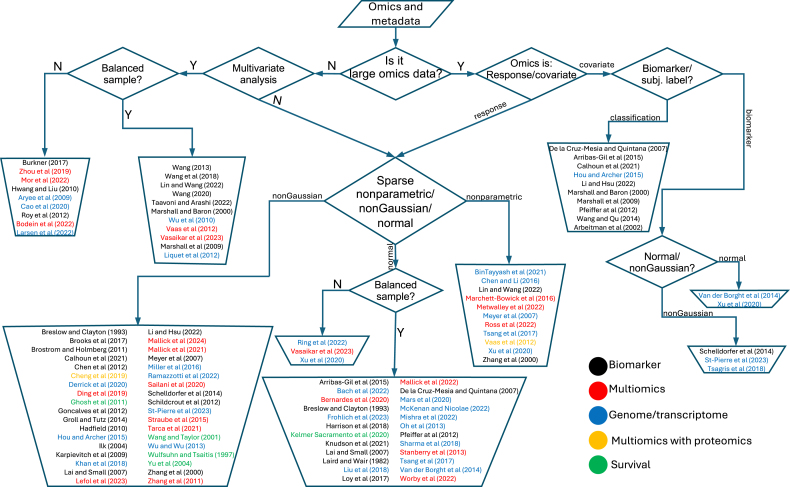
General step-by-step workflow for supervised analysis of longitudinal omics data (LOD). The workflow highlights multivariate methods applicable to low- to moderate-dimensional omics settings, such as biomarker studies or targeted metabolomics, where increased statistical power can be achieved. It further distinguishes between studies in which omics measurements are modeled as response variables influenced by clinical or experimental metadata, and studies in which omics features are treated as covariates to explain or predict changes in phenotypic or clinical outcomes. The details of each entry are provided in the supplementary Section B in the supplementary material.

Addressing these challenges requires robust statistical methods, computational advancements, and refined study designs, which we discuss in subsequent sections. A brief list of reviewed studies implemented supervised methods in the analysis of LOD are summarized in a step-by-step flowchart in Fig. 2 (see also Algorithms A1 and A2 in the supplementary materials).

## 2 LMM-based approaches for longitudinal analysis of omics

LMM/GLMM provide a foundational framework for longitudinal omics analysis by accounting for within-subject correlation under repeated measurements (see Table 1 for a comparative overview). However, classical formulations alone do not fully address two defining challenges in longitudinal omics studies: (i) high-dimensionality of molecular features relative to sample size and strong cross-feature correlation, and (ii) integration of multiple omics layers measured over time. Accordingly, we briefly introduce the standard mixed model notation to establish terminology and then emphasize mixed model extensions that incorporate regularization, multivariate structures, and hierarchical modeling strategies that are more directly tailored to longitudinal omics settings (see Section C of the supplementary material). For a single omics feature, let $y_{i}$ represent the measurements (e.g., count, abundance, intensity) for the $i^{\text{th}}$ subject at time points $t_{i1},\ldots ,t_{in_{i}}$, with a corresponding design matrix $X_{i}$ for fixed effects. Subject-specific random effects, $b_{i}$, account for intra-subject correlations via a known design matrix $Z_{i}$:

(1) $$y_{i}=X_{i}\beta +Z_{i}b_{i}+\varepsilon_{i},$$

where $\varepsilon_{i}\overset{iid}{∼}N(0,\Sigma_{\text{Err}})$ is Gaussian noise, independent of $b_{i}\overset{iid}{∼}N(0,\Sigma_{\text{rndEff}})$. Estimation via restricted maximum likelihood (REML) is widely available (e.g., lme4 [16], nlme [17], and proc mixed in SAS). Inflation in the false positives and bias in the inference [18] are the common side effects of ignoring subject-level correlation (i.e., omitting random effects) in omics data practices.

**Table 1:** Decision-oriented comparison of major longitudinal omics analysis method families. Each row summarizes the primary modeling goal, key statistical assumptions, strengths and limitations with respect to high-dimensionality, irregular sampling, missingness, nonlinearity, and compositionality, as well as computational demands and software maturity. This table is intended as a practical guide to support method selection based on data characteristics and scientific objectives in longitudinal omics studies.

Method family	Primary goal	Best-fit data settings	Pros	Cons	Software/codes
**LMM**	Mean trajectory inference; fixed effects (time, treatment, interactions), random effects (subject, nest); feature-level effect sizes/p-values	Continuous outcomes (e.g., log-intensity), modest number of time points; repeated measures; interpretable covariates	Interpretability; handles within-subject correlation via random effects; standard inferential workflow; good default baseline; Bayesian inferences have been well-developed	Normality and linearity assumptions may be too restrictive; high-dimensional feature-by-feature multiplicity can underfit nonlinear biology; not inherently designed for joint multiomics modeling	lme4, nlme; often used as baseline comparator
**GLMM and zero-inflated**	Mean trajectory inference for non-Gaussian outcomes; odds ratios; feature-level inference	Counts/relative abundance proxies; overdispersion/zero inflation common in microbiome and some sequencing summaries	Better suited than Gaussian models for counts; can include random effects and covariates; supports ordinal outcomes	Computation can be heavy; model misspecification (link, zero-inflation) impacts inference; compositionality still nontrivial	glmmTMB, MCMCglmm, brms; check convergence
**Population-averaged models (e.g., GEE)**	Marginal mean effects; robust SEs; average population trend	Moderate N, repeated measures; focus on mean trend rather than subject-specific trajectories	Robust to some misspecification; avoids full random-effect distribution assumptions; often stable in practice	Less subject-specific interpretation; correlation structure choice still matters; limited for complex missingness mechanisms	geepack; good sensitivity-analysis companion
**Semi-parametric (splines in mixed models)**	Flexible time trend estimation; smooth trajectories; feature clustering by fitted curves	Nonlinear biology with moderate time resolution; continuous outcomes (or via GLMM extensions)	Captures nonlinearity while keeping interpretability; can improve fit vs linear time	Knot/penalty tuning; may still be feature-by-feature; extrapolation unstable at edges	Spline LMM workflows; choose df/penalty carefully
**FDA**	Treat each subject feature-trajectory as a function; FPCA scores; functional regression; curve-level summaries	Dense or moderately dense time grids (or pre-smoothing); time-course-like designs also common	Strong for shape/phase variation; dimension reduction via FPCA; useful for classification and trajectory clustering	Needs smoothing/registration choices; irregular sparse sampling can be challenging; interpretability depends on basis/FPCA	FDA toolkits; useful when curves are meaningful objects
**GP/LonGP/waveome**	Nonparametric time modeling with uncertainty; kernel-based effects; hypothesis testing via kernel components	Irregular sampling; nonlinear dynamics; time-varying effects; moderate feature counts or careful screening	Flexible trajectories; principled uncertainty; kernels handle periodicity/long-range dependence; good for irregular schedules	Scalability limits in high-dimensional settings; kernel selection/model selection complexity; compute/memory demands	LonGP/GPcounts/waveome; consider approximation/feature screening
**High-dimensional regularized regression (penalized)**	Feature selection; predictive signatures; sparse time-varying effects (sometimes)	Large number of features, smaller sample size; moderate time points; goal is parsimonious biomarkers	Controls overfitting via regularization; yields sparse signatures; can be extended to longitudinal structure	Stability depends on tuning; may ignore temporal correlation if naïvely applied, no universal method for the hyperparameter values such as the penalty term coefficients	glmnet, glmmLasso
**Longitudinal/time-course DEA**	Identify features changing over time or differing trajectories across conditions; FDR-controlled feature lists	Transcriptomics and similar high-throughput, repeated measures or time-course designs	Directly answers “what changes”; integrates time trend (sometimes nonlinear); supports pathway follow-up	Methods vary widely (parametric/nonparametric/Bayesian); design mismatch (time-course vs longitudinal) can confuse; confounding and batch effects are critical	Organize as: mixed-model DEA, spline-based DEA, Bayesian time-course DEA, pathway-level DEA
**Multiomics integration (mixed-model, joint, network)**	Joint longitudinal inference across omics layers; cross-omics associations with subject-level effects	Repeated multi-layer measurements; heterogeneous scales; missing layers; often small sample size	Preserves temporal structure via random effects; improves interpretability across omics; supports mechanistic insight	Computational complexity; temporal alignment across layers; scalability in high-dimensions	Mixed-effect multiomics models; joint regression and network-based frameworks
**Clustering/trajectory grouping/networks**	Discover latent trajectory groups; co-dynamics modules; interaction networks over time	Exploratory settings; moderate time points; often used for mechanism discovery	Useful unsupervised structure discovery; generates interpretable modules; supports downstream enrichment	Results sensitive to distance/smoothing choices; stability/replicability can be weak; fewer mature longitudinal-omics-specific tools	Report robustness checks and cluster stability
**Survival/dropout/joint longitudinal–survival**	Time-to-event prediction; handle informative dropout; dynamic risk using longitudinal signals	Clinical cohorts with dropout/mortality; when event time is central outcome	Addresses informative missingness via joint modeling; interpretable hazard effects; principled handling of dropout	Model complexity and computation; event sparsity; requires careful assumptions and diagnostics	Joint models (LMM + Cox-style) are common; clearly state missingness mechanism
**Classification (time-aware discriminants, RF, etc.)**	Predict class/phenotype using longitudinal profiles; misclassification error, AUC	Small-to-moderate sample size; repeated measures; when prediction is primary goal	Can exploit longitudinal structure (time-aware discriminants); sometimes strong accuracy on benchmark designs	Risk of leakage if time/subject split mishandled; interpretability varies; some RF variants ignore time effects	Time-aware discriminant/classifier families; always define evaluation protocol
**Deep learning (RNN/LSTM/GRU, attention/Transformers)**	Flexible sequence modeling; representation learning; prediction/imputation; dynamic network inference	Large datasets or multi-cohort training; irregular sampling (with attention/time encoding); multimodal inputs	Models nonlinear interactions and complex temporal dependence; handles multimodal features; can support imputation	Data-hungry; compute-heavy; interpretability limited; risk of overfitting in typical omics sample sizes	Position as complementary; highlight hybrid/physics/statistics-informed designs

Mixed models are widely used in longitudinal biomedical studies [19] and remain central in longitudinal omics because they provide an inferential framework for separating systematic effects (e.g., time, treatment) from subject-specific variation. In longitudinal omics settings, practical extensions are often required to handle non-Gaussian outcomes, missingness and imbalance, high-dimensional feature spaces, and multiomics integration, which we discuss in the following subsections.

### 2.1 Linear mixed models: omics features under the normality assumptions

Alongside exploratory analysis [20], continuous omics features are commonly modeled using LMM, as seen in bulk and single-cell transcriptomics and proteomics studies [9], [21], [22], [23], [24]. LMM partitions covariate effects into fixed effects (systematic influences) and random effects (within-subject correlation, e.g., patient- or site-specific variability). Under approximate normality, model (1) supports inference on time and metadata effects while accounting for repeated-measures dependence.

Standard LMM assumptions and diagnostics apply [25], but in high-dimensional omics analyses they are often applied in a feature-wise manner with limited per-feature sample sizes, motivating shrinkage, regularization, and careful multiple-testing control.

For example, a longitudinal transcriptomic analysis of LINE-1 activity in Parkinson’s disease analyzed 423 patients with up to five follow-ups using feature-wise LMMs with subject random effects [24]. Each response was analyzed via LMM, with p-values adjusted using the Benjamini-Hochberg method. A similar approach was applied to longitudinal gut microbiome data [26]. The application of LMM on biosynthetic gene clusters (BGCs) can be found in BGC reviews [27] and meta-analysis [28].

For greater flexibility, spline bases can replace squared time terms [29] in LMM. This involves filtering low-variance genes, regressing each feature on sampling time using spline LMM where $f(t_{ij})$ replaces $\beta_{0}+\beta_{1}t_{ij}$ and performing clustering and differential expression analysis based on the best-fit model. This mixed model spline backbone is also commonly used as a first-stage trajectory estimation step in downstream clustering and multiomics integration pipelines (see e.g., Section 2.5).

**Fig. 3:**
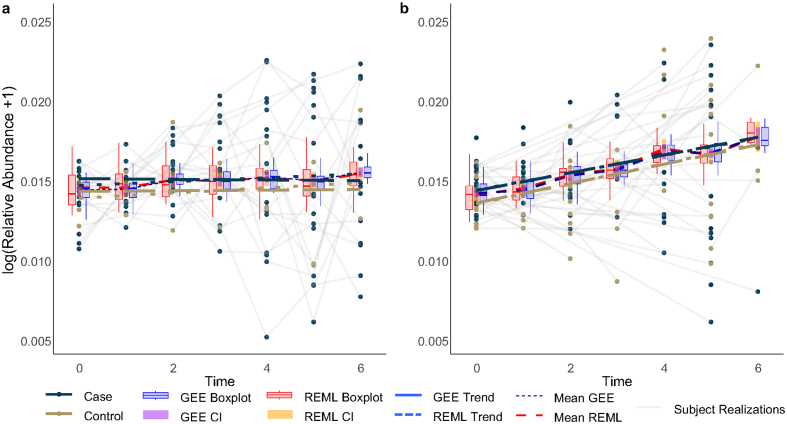
The performance of REML and GEE for LMM is compared for **a**, balanced data from 20 subjects observed at seven time points, and **b** imbalanced data of the same size but with varying observation times per subject. Treatment groups are distinguished by navy blue (case) and khaki (control). Mean fitted value curves are shown as dashed blue (GEE) and red (REML) lines. The dynamics of log⁡(relative abundance+1) over time are represented by dashed (GEE) and dotted (REML) lines for each group. Boxplots of fitted values appear at each time point in transparent blue (GEE) and red (REML), with 95% confidence intervals (CIs) marked by purple (GEE) and orange (REML) rectangles. Greater variation and longer, uneven CIs in the imbalanced case highlight the need to assess data balance before applying these methods. (For interpretation of the references to colour in this figure legend, the reader is referred to the web version of this article.)

To quantitatively assess the impact of sampling imbalance, we conducted a simulation study with 200 replications comparing the estimation accuracy of the time effect under balanced and imbalanced designs. For the LMM, the absolute bias of the estimated time coefficient increased by approximately 43% under imbalance (|−0.0259| versus |−0.0173|), and the mean squared error (MSE) increased by a factor of 2.66 (0.104 versus 0.039). Similarly, for GEE (geepack), imbalance led to a 66% increase in bias magnitude and a 2.64-fold increase in MSE. Coverage of nominal 95% confidence intervals was also affected, decreasing from 0.94 to 0.93 for GEE and from 0.89 to 0.93 for LMM, highlighting the sensitivity of inference to sampling imbalance. These quantitative results corroborate the visual discrepancies observed in Fig. 3. In one-time replication, this figure shows that the linear mean estimation curves at endpoints and fitted value boxplots differ from the balanced case. The imbalance also widens confidence intervals (CIs). In the balanced case (Fig. 3**a**), GEE minimizes variance, making boxplots and CIs nearly invisible, whereas precision deteriorates in the imbalanced scenario.

### 2.2 Abundances and non-Gaussian relative abundances

Many longitudinal omics measurements, particularly sequencing-based abundances, violate Gaussian assumptions due to discreteness, skewness, overdispersion, and zero inflation. Generalized linear mixed models (GLMMs) [30] extend LMMs by linking the expected response to fixed and random effects through a nonlinear function:

(2) $$E[y_{i}|b_{i}]=g^{-1}(X_{i}\beta +Z_{i}b_{i}),$$

where $g^{-1}(\cdot )$ is the inverse link function and y|b follows an exponential dispersion family distribution. Although GLMMs are well suited for longitudinal count and compositional data, their computational cost has limited widespread adoption in large-scale omics analyses. Nevertheless, several implementations support zero inflation and overdispersion, including glmmTMB [31], glmmML [32], and Bayesian frameworks such as MCMCglmm [33] and brms [34].

LOD primarily relies on feature abundances, often transformed into relative abundances. However, normal approximations fail for skewed distributions, and Poisson-based models require high intensity. Overdispersion and zero inflation further challenge normality assumptions, necessitating discrete distributions. Standard GLMM tools (e.g., glmm [35] package in R) model binomial and Poisson distributions, while Maaslin2 [36] incorporates compound Poisson and zero-inflated negative binomial models.

Zero-inflated beta regression model [37] is a suitable GLMM for longitudinal gut microbiome data. In a study on pediatric IBD, repeated measurements were collected from the same subjects over time (longitudinal repeated-measures design) from 47 children receiving anti-TNF therapy and 12 undergoing exclusive enteral nutrition (EEN) at baseline, one, four, and eight weeks. Relative abundances at the genus level were analyzed to identify treatment-associated bacterial changes over time. Let $y_{ig}(t_{ij})$ denote the relative abundance of an omics feature g for subject i at time $t_{ij}$ where it is zero with probability $p_{ig}(t_{ij})$ and distributed as $Beta(\mu_{ig}(t_{ij})\phi_{g},(1-\mu_{ig}(t_{ij}))\phi_{g})$ with probability $p_{ig}(t_{ij})$. The regression models are:

(3) $$\text{logit}(p_{ig}(t_{ij}))=\alpha_{0}+X_{ij}\alpha +\varepsilon_{ij},$$

(4) $$\text{logit}(\mu_{ig}(t_{ij}))=\beta_{0}+Z_{ij}\beta +\epsilon_{ij},$$

where ε and ϵ are white noise, and X and Z denote fixed and random effects. Parameter estimation is performed via maximum likelihood. This formulation illustrates how GLMMs can be tailored to longitudinal omics by explicitly modeling excess zeros, bounded supports, and subject-level heterogeneity within a unified inferential framework.

### 2.3 Compositionality in longitudinal omics data

Many omics platforms, including microbiome, metabolomics, and lipidomics, generate compositional data in which observed abundances are constrained to a constant sum. Ignoring this constraint can induce spurious correlations and misleading temporal patterns. A common strategy is to apply Aitchison log-ratio transformations [38] (e.g., centered or additive log-ratios), which map compositional data to an unconstrained space prior to longitudinal modeling. However, zero values complicate log-ratio approaches, raising an important distinction between true biological absence and technical zeros arising from detection limits. As a result, longitudinal compositional analyses often rely on two-part or zero-inflated mixed models that separately model presence and relative abundance, or on compound Poisson and beta-based formulations that partially respect compositional structure while accommodating sparsity. Developing scalable longitudinal models that jointly respect compositional constraints, zero inflation, and subject-level correlation remains an open challenge.

### 2.4 High-dimensionality of omics data

A defining challenge of longitudinal omics analysis is the high-dimensionality of molecular features relative to sample size, which renders classical mixed-model estimation unstable without additional structural assumptions. Adaptations, such as the CBCV-CorrConf algorithm [39] (available in R package CorrConf), address this by detecting significant random effects and estimating their influence on correlation structures. This method, validated on sex-specific DNA methylation in a 15 twin-pair study [40], identified 38% of known sex-associated methylation sites among 330,168 examined, aligning with findings from the longitudinal birth cohort CHAMACOS study [41]. To address this challenge, mixed-model-based approaches have been adapted through regularization, shrinkage, and feature selection mechanisms that preserve subject-level correlation while enabling inference in high-dimensional settings.

Dimension reduction techniques, including tensor factorization [42], have been used in longitudinal proteomics. Alternatively, fitting separate LMMs for each feature, followed by FDR correction, ensures nominal significance, albeit at the cost of testing power. Regularization methods, such as glmmLasso [43] and glmmixedlasso [44], aid in variable selection by minimizing a penalized log-quasi likelihood function. These approaches have been applied to gene expression data to track scarring progression over 11 time points [45]. Faster alternatives include MXM [46] in R and PenalizedGLMM.jl [47] in Julia, which accommodate between-individual correlations and binary traits.

An extension of LMM involves evaluating a set of multiple LMMs with predefined variables using multi-model inference and a genetic algorithm [48]. In a study on RAL resistance due to mutations in the HIV integrase region, 153 subjects with an average of three repeated measurements and 991 clonal genotype–phenotype pairs were analyzed. The study compared OLS and LMM-based variable selection using a genetic algorithm. For discrete omics data, penalized GLMMs extend to ordinal response variables, employing forward stagewise algorithms [49] to identify predictive gene sets in a longitudinal microarray dataset of inflammation and injury response studies.

Longitudinal studies often face challenges due to non-random sampling, particularly when costly biomarkers are involved. For example, a biased sampling design [50] was used in a study where daily urine samples determined the optimal day for serum sampling, which coincided with a surge in luteinizing hormone (LH). The study assessed the effect of fiber intake on LH surge probability, introducing bias via an auxiliary variable, modeled as follows:

$$\begin{array}{ll} & P({\text{LH}}_{ij}>20|X_{ij},\text{having serum sample for subject }i\text{at time }j)\\ & \;={\text{logit}}^{-1}(\log[\frac{P(\text{having serum sample for subject }i\text{at time }j|X_{ij},{\text{LH}}_{ij}>20)}{P(\text{having serum sample for subject }i\text{at time }j|X_{ij},{\text{LH}}_{ij}\leq 20)}]\\ & \;+X_{ij}\beta ). \end{array}$$

A random-effects version of this model for analyzing longitudinal binary data with biased sampling is discussed in [50]. The observation-dependent samplings are less expensive, and the probability of inclusion in the sample comes from auxiliary variables. A sequential offset logistic regression models the response variable onto the auxiliary variables and then the auxiliary variables onto the metadata. This can adjust the imbalance induced by the rarity of a phenotype, e.g., $P({\text{LH}}_{ij}>20)\approx 0$ or 1, marginally. These regularized mixed-model formulations are particularly well suited for longitudinal omics, where thousands of features are modeled in parallel under shared experimental designs.

### 2.5 Mixed-model approaches for multiomics longitudinal integration

While classical LMM and GLMM formulations are typically applied to a single omics layer at a time, recent methodological developments have extended mixed-model frameworks to enable the joint analysis of multiple longitudinal omics modalities. These approaches explicitly model subject-specific heterogeneity and temporal dependence while integrating heterogeneous molecular layers such as the microbiome, metabolome, transcriptome, epigenome, and proteome.

Early applications of longitudinal multiomics integration primarily relied on hierarchical mixed-effect models to disentangle within-subject temporal dynamics from between-subject variability. A prominent example is the longitudinal multiomics study of irritable bowel syndrome by Mars et al. [51], which jointly analyzed microbiome composition, microbial metabolites, host transcriptomics, and methylation profiles collected repeatedly over time. Linear mixed models were used to quantify temporal variability and subtype-specific effects, enabling the identification of microbial–metabolite–host interactions that were not detectable in cross-sectional analyses. Similar mixed-effect strategies have been employed in longitudinal inflammatory bowel disease cohorts [52], where repeated multiomics measurements were integrated to link microbial dynamics with host immune and metabolic responses over time.

Beyond application-driven studies, several methodological frameworks have been developed to support mixed-model-based multiomics integration in high-dimensional longitudinal settings. Multivariate and penalized mixed models have been proposed to jointly model multiple omics features while accounting for temporal correlation and subject-level random effects [37], [53]. These methods leverage regularization or low-rank structure to address the curse of dimensionality and enable stable estimation across thousands of molecular features. Related approaches incorporate variance-component testing and hierarchical modeling [54], [55] to identify cross-omics associations that persist over time rather than at isolated time points.

More recently, integrative mixed-model frameworks have been extended to accommodate complex experimental designs and heterogeneous data types, including uneven sampling schedules and modality-specific noise structures. For example, recent longitudinal multiomics models combine random effects with latent factor representations to capture shared temporal trajectories across omics layers while preserving modality-specific signals [56]. These approaches bridge classical mixed models and modern multivariate learning, providing a statistically principled foundation for longitudinal multiomics integration.

Overall, mixed-model–based multiomics methods represent a natural extension of traditional longitudinal modeling, offering interpretable inference, explicit handling of repeated measurements, and robustness to unbalanced designs. However, their scalability and computational complexity remain challenging as the number of omics layers and features increases, motivating ongoing work that combines mixed models with dimension reduction, Bayesian shrinkage, and hybrid machine learning approaches. Table 1 summarizes these mixed-model-based longitudinal multiomics approaches alongside classical LMM/GLMM formulations, highlighting their relative strengths in handling high-dimensionality, multiomics integration, interpretability, and unbalanced longitudinal designs.

A central difficulty in longitudinal multiomics integration lies in reconciling heterogeneity across data layers. Omics modalities often differ in temporal resolution and sampling frequency, leading to temporal misalignment that complicates joint modeling. In addition, layers vary substantially in measurement noise, dynamic range, and feature scale, making naive concatenation or joint normalization problematic. Current approaches, therefore, adopt different integration strategies, ranging from subject-level integration (e.g., shared random effects or latent trajectories across omics layers) to feature-level integration (e.g., cross-omics association or network analyses). While subject-level approaches preserve longitudinal structure, they often sacrifice molecular specificity, whereas feature-level approaches scale poorly and are rarely extended to fully joint longitudinal interaction models.

### 2.6 Differential expression analysis

Differential gene expression analysis (DEA) aims to identify molecular features whose expression profiles vary across conditions or over time. In LOD, the primary challenge lies in modeling dynamic expression changes while accounting for repeated measurements and within-subject correlation. In contrast, time-course experiments typically involve population-level sampling across time points without subject-level replication, leading to distinct analytical requirements.

#### 2.6.1 Time-course versus longitudinal designs in DEA

Although the terms *time-course* and *longitudinal* are sometimes used interchangeably in the omics literature, this distinction is particularly important for DEA and has broader implications throughout longitudinal omics modeling.

In a time-course study, molecular measurements are collected across multiple time points but typically without repeated measurements on the same experimental units (or with limited replication). Such designs are common in controlled experiments (e.g., cell lines or animal models) and are often analyzed using smoothing, clustering, or functional approaches that emphasize overall temporal patterns rather than subject-specific effects.

In contrast, a longitudinal study involves repeated measurements on the same individuals or biological units over time, inducing within-subject correlation that must be explicitly modeled. Longitudinal DEA therefore requires statistical frameworks such as mixed-effect models, generalized estimating equations, or joint longitudinal models that account for subject-specific variability, imbalance in follow-up times, and missing observations.

As a practical example, bulk RNA-seq measured at predefined time points from independent samples constitutes a time-course design, whereas repeated transcriptomic profiling of the same patients across clinical visits represents a longitudinal design. Throughout this review, we explicitly indicate whether cited studies correspond to longitudinal repeated-measures designs or time-course experiments to avoid conceptual ambiguity.

#### 2.6.2 Mixed-model–based longitudinal DEA

For longitudinal designs, standard generalized linear models must be extended to account for within-subject dependence. Mixed-effect models achieve this by incorporating subject-specific random effects, allowing inference on fixed effects such as time, treatment, and their interactions. Ignoring subject-specific effects can inflate false positives, particularly when samples are imbalanced across time points.

Generalized linear mixed models (GLMMs) are widely used for longitudinal RNA-seq and other count-based data, often assuming negative binomial or related distributions. Extensions link variance to the mean via dispersion parameters from the exponential dispersion family, enabling flexible modeling of overdispersion. Such frameworks can also be applied to relative abundances with appropriate distributional assumptions (see Supplementary material, Section E).

#### 2.6.3 Nonparametric and spline-based time-course DEA

For time-course experiments without subject-level replication, nonparametric approaches are commonly employed. Wu and Wu [57] proposed a functional principal component–based framework for time-course gene expression data, modeling $y_{igc}(t_{ij})=f_{gc}(t_{ij})+\varepsilon_{ij}$, testing

$$\begin{array}{l} \left\{ \begin{array}{ll} H_{g0}:f_{gc}(t)=f_{g}(t) & \text{for all }t\text{and }c,\\ H_{g1}:f_{gc}(t)\neq f_{gc^{\prime }}(t) & \text{for some }c,c^{\prime }, \end{array}\right. \end{array}$$

for subject i, feature g, and condition c. Rejection of $H_{g0}$ identifies temporally regulated biomarkers while controlling false discovery rates.

Spline-based regression approaches similarly model smooth temporal trajectories and are frequently used in balanced time-course designs. In these settings, classical DEA tools often treat time points as categorical conditions.

#### 2.6.4 Bayesian time-course DEA

Bayesian formulations provide a principled framework for modeling uncertainty in time-course DEA. Cao et al. [58] proposed a Bayesian negative binomial model for time-course RNA-seq data:

(5) $$E[y_{igc}(t)|\mu_{igc}(t)]=S_{gc}\exp\{\theta_{g0}1_{\left\{c=1\right\}}$$

(6) $$\;+B^{⊤}(t)\theta_{g1}1_{\left\{c=1\right\}}+B^{⊤}(t)\eta_{g}\},$$

where B(t) denotes spline basis functions, 1 is the indicator function and $B^{⊤}$ is the transpose of B. Hypothesis testing distinguishes condition-specific effects from time-varying effects, implemented in the MAPTest package. Alternative autoregressive tests [59] for temporal effects have also been proposed.

Although some longitudinal omics studies do not explicitly use Bayesian DEA models, tools such as DESeq2 [60] rely on Bayesian-inspired generalized linear modeling and empirical Bayes shrinkage, and have been applied in both bulk and single-cell transcriptomic studies [61], [62]. However, there is still need to take care of the within-subject correlation in using DESeq2 for longitudinal data.

#### 2.6.5 Pathway-level longitudinal DEA

Beyond gene-level inference, longitudinal DEA is often conducted at the pathway level to improve interpretability and robustness. In metabolomics studies, pathway-based methods compute aggregated expression summaries across time points and assess temporal changes in pathway activity. Stanberry et al. [63] and Haynes et al. [64] proposed approaches that identify temporally enriched subpathways by weighting metabolite-level signals and evaluating their longitudinal influence.

#### 2.6.6 DEA in single-cell and high-throughput longitudinal settings

Single-cell and high-throughput longitudinal studies introduce additional challenges, including sparsity, technical noise, and complex dependency structures. Pipelines such as TiSA [65] integrate time-course DEA using DESeq2 or limma, followed by clustering and pathway enrichment. While such tools are primarily designed for time-course data, they are frequently applied to longitudinal studies by aggregating observations across time points. A broader list of Bayesian and longitudinal DEA tools is provided in Section G of the supplementary material.

### 2.7 Longitudinal single-cell omics data analysis

In contrast to bulk and other aggregated omics data, longitudinal single-cell studies rarely employ classical LMM or GLMM frameworks; instead, they predominantly rely on cell-state classification, trajectory inference, and clonal or lineage tracking, reflecting a fundamental mismatch between subject-level repeated-measures models and the dynamic, non-exchangeable nature of single-cell observations.

Single-cell omics technologies provide an unprecedented view of cellular heterogeneity and dynamic state transitions in complex biological systems. When combined with temporal sampling, they offer the potential to track how cell populations evolve during disease progression, treatment, or immune response. However, despite the frequent use of the term *longitudinal* in the single-cell literature, true longitudinal single-cell omics studies defined as repeated measurements from the same individuals over time remain relatively rare, particularly in human cohorts.

Many studies labeled as longitudinal single-cell analyses are, in fact, *time-course* experiments, where samples collected at different time points originate from different individuals or sacrificed model organisms. For example, longitudinal single-cell transcriptomic studies of inflammatory bowel disease models in mice often profile tissue at successive disease stages but do not involve repeated measurements from the same animal, and thus represent time-course designs rather than subject-level longitudinal data [66], [67]. These studies primarily address how cellular composition and transcriptional programs change across disease progression rather than modeling within-subject temporal dependence.

In contrast, a smaller but growing body of work has leveraged true longitudinal single-cell designs in human studies, where repeated samples are collected from the same individuals across clinically meaningful time points. Such designs are common in cancer and immune-related diseases, where serial biopsies or blood draws are feasible. For instance, longitudinal single-cell RNA-sequencing studies in acute myeloid leukemia and ovarian cancer explicitly compare diagnosis versus relapse or pre- versus post-treatment samples within the same patients, enabling direct characterization of transcriptional state transitions associated with disease recurrence or therapeutic resistance [68], [69]. Similarly, longitudinal profiling of antigen-specific B cells following SARS-CoV-2 infection [70] tracks clonal persistence and transcriptional evolution across acute and convalescent phases using repeated sampling from the same individuals.

Across these true longitudinal single-cell studies, the dominant analytical focus is not classical differential expression testing but rather *classification* and *state identification*. Most analyses aim to determine whether cells transition between transcriptional states, lineages, or functional phenotypes over time, and whether these transitions differ across clinical outcomes such as relapse, severity, or treatment response. Accordingly, commonly used statistical and computational tools include clustering, low-dimensional embedding, pseudotime or trajectory inference, and cell-type proportion analysis, often followed by differential expression or pathway enrichment within identified states. Explicit mixed-effect or joint longitudinal models are rarely employed, largely due to the extreme dimensionality, sparsity, and computational burden of single-cell data.

Several large-scale human studies exemplify this paradigm. Longitudinal single-cell atlases of COVID-19 patients, constructed from repeated blood samples, focus on the temporal reorganization of immune cell states and the timing of interferon responses relative to disease severity [71]. In chronic myeloid leukemia, longitudinal single-cell transcriptomics has been used to characterize the evolution of micro- and macro-cellular states over time, framing disease progression as transitions among relatively stable transcriptional attractors rather than continuous trajectories [72]. These studies underscore that, in practice, longitudinal single-cell analysis often reduces to a classification or state-transition problem under temporal constraints.

Methodological work has also emerged to support longitudinal single-cell study design and benchmarking. Simulation frameworks such as RESCUE and rescueSim explicitly generate paired and longitudinal single-cell RNA-sequencing data with controlled temporal and subject-level structure, enabling evaluation of downstream methods under realistic noise and dependency patterns [73], [74]. While these tools do not analyze empirical cohorts, they highlight the growing recognition of longitudinal dependence as a critical feature in single-cell data generation and analysis.

Longitudinal single-cell omics usually occupies a distinct methodological niche within longitudinal omics analysis. Human longitudinal data remain limited due to cost, tissue accessibility, and ethical constraints, and most existing studies prioritize classification, state dynamics, and trajectory characterization over formal longitudinal inference. Developing statistically principled models that can jointly account for subject-level dependence, temporal structure, and high-dimensional single-cell measurements, while remaining computationally tractable, remains an open and important challenge for the field.

## 3 Modeling sample paths

### 3.1 Functional data analysis

Longitudinal trajectories can be modeled as functions of time or continuous covariates, assuming $y_{ij}∼N(f(t_{ij}),\sigma^{2})$. A common approach is cubic spline estimation with smoothness constraints [10], facilitating visualization and hypothesis testing for group differences (e.g., case vs. control). This requires sufficient repeated measurements per subject. The OmicsLonDA package provides an accessible implementation for LOD analysis.

A stochastic mixed model incorporates dependence structures and metadata effects:

$$\begin{array}{l} y_{ij}=X_{i}\beta +f(t_{ij})+b_{i}+U_{i}(t_{ij})+\varepsilon_{ij}, \end{array}$$

where f is a smooth function of time, $b_{i}∼N(0,\phi )$ captures subject-specific intercepts, and $U_{i}(t)$ is a Gaussian process with correlation function $\eta_{\rho }(t,s)\equiv \text{Corr}(U_{i}(t),U_{i}(s))$ for the correlation parameter ρ. This model has been used to study progesterone levels across menstrual cycles while adjusting for BMI and age [75]. A simplified functional regression model removes random effects [76], applied in the Study of Women’s Health Across the Nation (SWAN) dataset to analyze menstrual cycle hormone levels with B-splines, revealing inflection points at the luteal transition [77].

Another FDA approach models longitudinal phenotypes, y, against genetic markers [78]:

$$\begin{array}{l} y_{ij}=f_{0}(t_{ij})+\sum_{g=1}^{G}\sum_{\gamma =0}^{2}f_{g}^{\gamma }(t_{ij})X_{g}^{\gamma }+\varepsilon_{ij}, \end{array}$$

where $f_{0}$ is the intercept function, $f_{g}^{\gamma }$ represents genotype-specific genetic effects, and G denotes the number of genes. This high-dimensional (only one of the terms$f_{g}^{0}X_{g}^{0}+f_{g}^{1}X_{g}^{1}+f_{g}^{2}X_{g}^{2}$ is nonzero per observation) genome-wide association studies (GWAS) model is implemented in Time-Varying Group SpAM code in Matlab [78] and has been applied to GWAS asthma studies.

Interdomain Gaussian Process (GP) modeling treats time-dependent responses as a GP realized at sparse points, inferring posterior distributions across domains. Formally, for subject i:

$$\begin{array}{l} y_{i}|X_{i},t_{i}\overset{ind}{∼}N(f(X_{i}),\sigma^{2}),\;f∼G(0,k(\cdot ,\cdot )), \end{array}$$

where k is a kernel function capturing intra-subject dependence, estimated using predefined structures. LonGP [79] extends this by decomposing f into additive components with different kernels, supporting various covariate types. The provided Matlab-based code supports squared exponential and periodic kernels for continuous covariates, as well as constant, binary, and categorical kernels for factors. For model selection, waveome [80] employs BIC with a more flexible model that includes both summation and multiplication of kernel functions, while LonGP uses leave-one-out and stratified cross-validation with Bayesian bootstrap.

LonGP [79] analyzed 758 stool samples from 222 children (birth to age three) located in three countries [81] to assess socioeconomic effects on the gut microbiome, identifying reduced microbial enrichment in Russian children. Applied to plasma proteomics from T1D and control children, it identified 38 disease-associated proteins, including 18 missed by LMM [82]. While LonGP assumes normal and Poisson models, GPcounts [83] extends it to negative binomial and zero-inflated models.

### 3.2 Low/high replication

Longitudinal studies leverage high replication and regularity to mitigate sparsity issues [84]. Phenotype microarray data, used to characterize microbial growth under specific conditions, offer balanced measurements but pose computational challenges for LMMs due to high-dimensional covariance structures. Curve fitting methods, such as cubic splines via smooth.spline() in R, provide a computationally efficient alternative. A study on 2 microbial species (*E. coli* and *P. aeruginosa*) × 4 strains × 2 biological replicates × ten technical replicates × 96 substrates provided 7680 time-dependent measurements and instead of comparing model parameters, the estimated area under the curves (AUC) was compared as a growth summary [84]. However, OmniLog® software introduces estimation biases, neglecting part of the stored information.

For LOD with small sample sizes, frequentist methods are not recommended. However, Bayesian GLMMs [85], Kenward-Roger approximation (pbkrtest [86]), and multilevel modeling [87] remain viable approaches.

## 4 Longitudinal omics: few subjects, many features

Longitudinal omics, such as single-cell (sc) analysis, lag behind other omics due to limited samples and high-dimensional measurements. scRNA samples, typically from the same tissue, track treatment response or cancer evolution [88]. The fishplot method [89] visualizes cell population proportions over time but loses symmetry when plotting clonal frequencies. Some scDNA approaches adapt bulk sequencing methods. A study of 47 metastatic patients modeled detectable and undetectable mutations at each time point [90], using a two-stage mixed model for changes from baseline and a Cox model for survival analysis.

Another approach characterizes disease progression via candidate driver mutations. A time-dependent random matrix represents somatic mutations, accounting for false positives, false negatives, and missing data. Boolean matrix factorization estimates cell attachment and phylogenetic matrices, maximizing a likelihood function via MCMC. The longitudinal analysis of cancer evolution (LACE) algorithm [91], available in the R package LACE, tracks genotype prevalence and reconstructs clonal evolution. Unlike CALDER [92], which clusters mutations based on allele frequencies, LACE controls error rates via weighted likelihood maximization, incorporating uncertainty through sum-condition-based factorization (see e.g., [93]).

The PALMO pipeline enables longitudinal multiomics analysis, spanning single-cell and bulk data [94]. It includes: (1) Variance decomposition analysis (VDA), using LMM to partition variation; (2) Coefficient of variation profiling (CVP), distinguishing stable vs. oscillating bulk features; (3) Stability pattern evaluation across cell types (SPECT), the scRNA counterpart of CVP; (4) Outlier detection analysis (ODA), identifying weak intra/inter-donor correlations; and (5) Time course analysis (TCA), using MAST for differential expression analysis. PALMO also provides visualizations, including circos plots, CV heatmaps, and UMAP projections. The study analyzed 60 plasma and PBMC samples from six donors over 10 weeks, integrating plasma protein abundances, flow cytometry, scRNA-seq (24 samples from four donors), and scATAC-seq (18 of these 24). Seurat identified 31 cell types from 472,464 cells, with 11,191 highly expressed genes. VDA, treating donors and time points as random effects, revealed strong inter-donor variation in CBC, PBMC, and plasma proteins, identifying 75 proteins with greater intra- than inter-donor variability. Including cell type as a random effect in single-cell data showed stronger inter-cell type variation. CVP identified 413 longitudinal and 629 stable proteins, the latter as potential biomarkers. ODA flagged weak intra-donor correlations at week 6 in one subject. SPECT counted gene CV exceedances across 76 combinations of 4 donors and 19 cell types, detecting 700 super-variable, 2129 super-stable, 5750 variable, and 4004 stable (STATIC) genes, with STATIC genes as biomarker candidates.

## 5 Classification based on longitudinal omics data

Logistic regressions using GLMM are commonly employed for classifying LOD. For instance, using a GLMM for ordinal data, Hou and Archer [49] classified 657 buffy coat samples from burn injury patients hybridized to Affymetrix Human Genome U133 Plus 2.0 Arrays, identifying key genes associated with organ failure.

Linear Discriminant Analysis (LDA) is widely used in omics classification but struggles with time effects and imbalanced case-control ratios in rare disease studies. To address these, Longitudinal Discriminant Analysis (LonDA) [95] estimates group-specific parameter distributions over time, defining decision boundaries as:

(7) $$\begin{array}{ll} & \log\pi_{k}+\log f_{k}(y_{i};\phi_{k}(t))\\ & \;\;\;\;=\max_{j}\{\log\pi_{j}+\log f_{j}(y_{i};\phi_{j}(t))\},\;j=1,\ldots ,g. \end{array}$$

With Gaussian group densities and equal covariances, the boundaries remain linear. LonDA accounts for mean and variance heterogeneity across time, leading to four estimation scenarios [95] (see the supplementary material, Section F): homoscedastic, mean-heteroscedastic, variance-heteroscedastic, and fully heteroscedastic models. The model has been applied to classify pregnancy outcomes based on beta-subunit measurements [95] and later analyzed via Bayesian nonlinear classification [96], multivariate clustering [97], and bivariate modeling under missing data assumptions [98], emphasizing the need for time-aware classification.

The Quadratic Inference Function Classifier [99] (QIFC) efficiently handles small sample sizes and, unlike LonDA, omits full covariance estimation by modeling time dependencies via a QIF-based distance. After fitting a semiparametric model, it computes the distance of a data-point from each class and then assigns it to the closest. Applied to omics datasets, QIFC outperformed logistic regression, SVMs, and functional data classifiers, achieving a 5.3% misclassification rate on yeast (*Saccharomyces cerevisiae*) cell cycle data [100] (2467 genes, 79 time points) and 14% on *Drosophila melanogaster* data [101] (4028 genes, 70 time points).

**Fig. 4:**
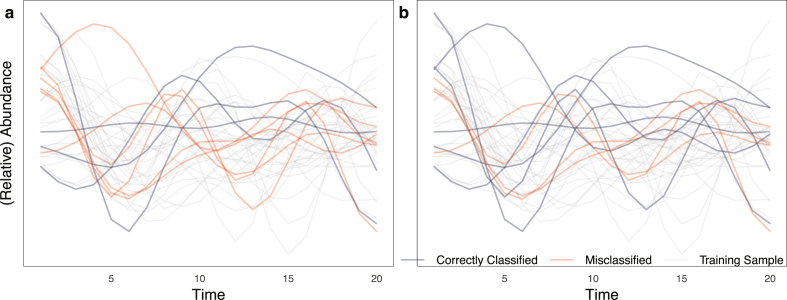
Comparison of **a** logistic regression and **b** Bayesian classifier [102] using m=30 subjects at $n_{i}=20$ time points, where 20 subjects were used for training and 10 for testing. Red lines indicate misclassified, and blue lines correctly classified samples. (For interpretation of the references to colour in this figure legend, the reader is referred to the web version of this article.)

FDA optimal Bayes classifier [102] offers a nonparametric classification approach by projecting time-dependent data onto specific directions (e.g., principal components). Applied to yeast data, this method achieved a 12.5% misclassification rate, outperforming logistic regression and centroid-based classifiers [103]. Fig. 4 compares logistic regression and the Bayesian functional classifier on sample data.

Regularization methods address the limitations of LDA in high-dimensional cases by estimating class-dedicated functions of time:

$$\begin{array}{l} y_{i}=X_{i}\beta +f_{k}(t_{i})+\varepsilon_{i}. \end{array}$$

Here, the nonparametric lasso estimate of $f_{k}$ is provided by a penalized EM algorithm [104].

Functional principal component (FPC) analysis has been used to classify Alzheimer’s patients based on 319 longitudinal biomarkers [105]. The FPC scores of subject i, $x_{i}$ are used to minimize the loss function

$$\begin{array}{l} l(\beta )=\frac{1}{N}\sum_{i=1}^{m}e^{-c_{i}x_{i}^{⊤}\beta }+\lambda \sum_{k=1}^{K}\sqrt{p_{k}}\|\beta_{k}{\|}_{2}, \end{array}$$

where $c_{i}$ is a binary disease class indicator, $\beta_{k}$ is the coefficient corresponding to the $k^{\text{th}}$ feature group, and $p_{k}$ is the number of FPC scores.

A distribution-free method [106] integrates longitudinal biomarkers via a composite marker score, ensuring no information loss in regression through sufficient dimension reduction. The method assumes decomposability of marker vectors into marker and time effects and models the response variable using a sufficient linear combination of markers, accounting for right censoring. Applied to a cancer cohort of 100 cases and 100 controls observed at 4 time points, logistic-type regression classified cases based on uric acid, blood glucose, and serum cholesterol measured at four time points.

Random forest (RF)-based classifiers are also widely used. RF++ [107] a library in C++ employs subject-level averaging and bootstrapping, successfully classifying 38 subjects (30 cancer cases and 8 controls), with substantial replication per subject. Despite subject-level averaging, it does not capture time effects [108]. Repeated-measures random forests have also been explored for time-aware risk stratification, for example classifying observation times into high-risk nocturnal hypoglycemia states using longitudinal metabolomics data [109].

## 6 Multivariate outcomes in clinical longitudinal studies

Omic data involves multiple correlated features measured simultaneously. While univariate analyses with multiple testing corrections (e.g., B-H) usually suffice [110], they fail to capture inter-feature dependencies, leading to high-dimensional GLMM challenges (see Section 2.4). Multivariate models offer a more holistic approach, particularly for the analysis of known biomarkers.

Consider p outcomes measured at $n_{i}$ time points for subject i=1,…,N, represented as $Y_{i}=[y_{i1}|\ldots |y_{ir}]$, an $n_{i}\times r$ matrix. The full dataset forms an $N\times r\times n_{i}$ array, with measurement errors $E_{i}=[\varepsilon_{i1}|\ldots |\varepsilon_{ir}]$.

The multivariate t linear mixed model (MtLMM) models the vectorized response $y_{i}=\text{vec}(Y_{i})$ as:

$$y_{i}=X_{i}\beta +Z_{i}b_{i}+E_{i},$$

where block-diagonal matrices $X_{i}$ and $Z_{i}$ allow different covariate sets per outcome. Measurement errors follow a $q_{.}+n_{i}$-variate central t distribution with scale matrix $\text{diag}\{D,R_{i}\}$ and ν degrees of freedom. The covariance structure $R_{i}=\Sigma_{\text{Err}}⊗C_{i}$ accommodates inter- and intra-subject correlations via a damped exponential correlation (DEC) function. Parameter estimation under missing at random and not at random employs the alternating expectation-conditional maximization algorithm [111] with Fisher scoring, and empirical Bayes shrinkage [6] estimates random effects.

Data from 161 women with 124 normal and 37 abnormal deliveries tracked estradiol and β-HCG levels during early pregnancy. Log-transformed responses were modeled with covariates (rescaled time, squared time, pregnancy group). AIC favored MtLMM with DEC, while BIC preferred AR(1); both dominated the normal LMM. MtLMM variants have also been extended to censored heavy-tailed [112] and missing data scenarios [113], [114].

The MtLMM was also applied to HIV-1 RNA counts in seminal and blood plasma from 149 subjects (106 on therapy, 43 not) with DEC, outperforming the normal LMM. Penalized likelihood extensions addressed high-dimensionality [115].

A constrained multivariate LMM analyzed BioCycle data [116] (259 women, ages 18–44), incorporating ground truth constraints (e.g., EC peaks before LH surge, followed by PG rise). The model for hormone h is

$$y_{ijh}=\mu_{jh}+X_{ij}^{⊤}\beta_{h}+\xi_{ih}+\zeta_{ij}+\varepsilon_{ijh},$$

enforces Aμ≤b for structured hormone progression where $\mu_{jh}$ is the marginal mean, $\xi_{ih}$ is the subject-specific random effect, and $\zeta_{ij}$ is the time effect. The constraint decreases bias by limiting the parameter space. An adaptive MCMC [117] estimates posterior summaries.

To aid practical method selection and provide a unified comparison across the diverse approaches reviewed here, Table 1 summarizes the primary modeling goals, key strengths, limitations, and suitable data settings for the major methodological classes used in LOD analysis. This table serves as a practical decision framework, directly comparing major methodological families across statistical assumptions, scalability, robustness to missingness and imbalance, computational demands, and software maturity, to guide method selection in longitudinal omics studies. Table 1 is designed to guide method selection rather than rank approaches. Readers should first identify their primary analytical goal (e.g., trajectory inference, feature selection, prediction, or integration), then assess key data characteristics such as sample size, number of time points, degree of imbalance or missingness, and dimensionality. For example, mixed-effect models provide interpretable inference for modestly sized longitudinal studies, while Gaussian process and deep learning approaches offer greater flexibility for irregular sampling at higher computational cost. No single method is universally optimal, and sensitivity analyses across complementary method families are often warranted.

## 7 Other scenarios

Some approaches do not fit neatly into one category but remain relevant to previous sections. We discuss them here.

### Deep learning

While earlier sections emphasize mixed-effect and model-based approaches due to their interpretability and maturity, this subsection focuses on state-of-the-art non-parametric and deep learning methods that are increasingly used to model high-dimensional, irregularly sampled LOD. Recent advances in deep learning have introduced flexible frameworks for modeling complex temporal dependencies in LOD. Recurrent neural networks (RNNs), including long short-term memory (LSTM) and gated recurrent unit (GRU) architectures, have been applied to capture nonlinear temporal patterns and delayed effects in high-dimensional molecular profiles, particularly in longitudinal microbiome and gene expression studies [118], [119]. More recently, attention-based and Transformer-inspired models have been explored in omics research to capture long-range dependencies and handle irregular sampling, although their application to explicit longitudinal omics trajectory modeling remains an emerging area [120].

In the context of omics data, deep learning approaches are particularly attractive for modeling nonlinear dynamics, complex feature interactions, and multivariate temporal dependencies. Applications include disease progression modeling, treatment response prediction, dynamic biomarker discovery, and temporally informed imputation of missing measurements [121]. Deep learning models have also been shown to recover dynamic interactions and regulatory relationships over time, for example, through inference of time-varying networks from time-series single-cell expression data [122]. In that study, the proposed approach was applied to three publicly available longitudinal scRNA-seq datasets, including a human SARS-CoV-2 vaccination time series, a human lung aging atlas from the Human Cell Atlas Project [123], [124], and a mouse lung injury model of pulmonary fibrosis [125]. Deep learning methods have further been used for missing value imputation in LOD [126] and for handling irregular sampling patterns [121]. However, these approaches typically require large sample sizes, careful regularization, and substantial computational resources, and they often provide limited interpretability compared with model-based statistical approaches [127], [128]. Consequently, deep learning methods are best viewed as complementary to traditional longitudinal models rather than replacements, particularly in biomedical settings where sample sizes are modest and interpretability is critical.

GRU architectures represent a streamlined variant of recurrent neural networks that are well-suited for modeling temporal dependencies in sequential data. In longitudinal omics studies, GRUs have been increasingly explored to capture time-dependent molecular patterns while mitigating some of the training and scalability challenges associated with more complex recurrent models. For example, DeepIDA-GRU integrates longitudinal and cross-sectional multiomics data using GRUs to jointly model temporal molecular trajectories and disease status [129]. The method was applied to longitudinal metagenomics and metabolomics data together with cross-sectional host transcriptomics from the iHMP inflammatory bowel disease cohort [130], with the goal of identifying temporally evolving molecular features that discriminate disease status while accounting for cross-omics associations. Similarly, MildInt [131] employs GRU-based encoders to integrate heterogeneous longitudinal and non-longitudinal modalities and was evaluated on multimodal longitudinal data from individuals with mild cognitive impairment in the Alzheimer’s Disease Neuroimaging Initiative cohort, combining neuroimaging, clinical, and demographic measurements to predict disease progression.

Ongoing research increasingly focuses on hybrid approaches that combine deep learning architectures with structured statistical modeling or network-based frameworks to improve robustness and biological interpretability in longitudinal omics analysis [119], [132].

### Integration

High-dimensionality is the key challenge in the integration of inferences based on multiple omics features from the same subject. For instance, in a study classifying subjects into type 2 diabetes and prediabetes using longitudinal transcriptomes, metabolomes, cytokines, and proteomes, intra-correlations were ignored by subtracting the mean of healthy time points from baseline measurements [9]. While this avoids estimating correlation effects, it sacrifices data granularity by summarizing with AUC values. There is also a network-based framework for integrating longitudinal multiomics data by combining data-driven inference with prior biological knowledge to construct hybrid multi-layer networks [133]. This approach emphasizes interpretability, using longitudinal clustering, enrichment analysis, and random-walk propagation to identify dynamic inter- and intra-omics interactions and regulatory mechanisms over time. The method is demonstrated on multiple real-world longitudinal case studies and is implemented in the R package netOmics, enabling exploration of temporal multiomics relationships beyond single-omics analyses.

Longitudinal multiomics data, particularly with small sample sizes are often analyzed feature-by-feature [134]. However, when data tables can be merged, high-dimensional methods are used, such as in studies of the gut microbiome, host transcriptome, and methylome in IBS patients [51].

The Bayesian Additive Regression Trees method integrates multiomics data in longitudinal studies via a two-stage modeling approach, as applied to the Integrative Human Microbiome Project [135]. To integrate longitudinal DNA methylation, mRNA, miRNA, and proteomics from developing murine alveoli, [136] introduced iDREM, which identifies transcription factors and miRNAs influencing gene expression via an input-output hidden Markov model combined with logistic regression [137].

### Clustering and abundance change network modeling

Clustering LOD remains an emerging field, with existing methods yet to be fully adapted [138], [139]. However, Larsen et al. [140] employed the clustering approach of Genolini et al. [141] to classify patients based on breast cancer LOD.

### ImpulseDEA

Longitudinal RNA and DNA methylome data from IBD patients were analyzed across two cohorts receiving anti-TNF therapy (infliximab or adalimumab) and a control cohort on vedolizumab [11]. Subjects were fully observed over time, with both pairwise time point comparisons and longitudinal approaches used to identify gene expression differences. The analysis employed the ImpulseDE2 model [142].

### Survival and dropout

In omics studies, particularly those involving severe diseases, subjects may leave before the study ends due to treatment changes, withdrawal, recovery, or death. When the primary focus is the time of exit (e.g., recovery/mortality), survival models are employed. While survival data is often sparse, some studies integrate longitudinal measurements to predict outcomes, such as CD4 counts in AIDS cohorts [143], [144] and PSA levels in prostate cancer [145]. Cox regression remains the dominant approach, either independently or within joint models [62] that assess the impact of longitudinal omics and baseline metadata on hazard rates.

A key advancement in joint modeling combines LMM with Cox regression to capture disease progression and dropout mechanisms. For instance, in a Zidovudine (ZDV) trial for HIV, CD4 counts served as surrogate markers for survival, with empirical Bayes estimates adjusting for missing data [143]. Extensions incorporated Bayesian inference and Ornstein-Uhlenbeck processes for time-varying coefficients, refining survival estimates [144]. Change-point models have also been applied to plasma HIV RNA data from AIDS trials, capturing virologic failure dynamics and dropout effects using Bayesian MCMC methods [146]. Given the predominant use of survival models in time-to-event analyses rather than longitudinal omics, a detailed discussion of these methods is provided in the supplementary material. More discussion on this topic is available in Section H of the supplementary material.

Finally, the topic of causality is of general interest in omics studies, where the goal is to transition from statements of association to causal effects. One line of research that attempts to address this is Mendelian Randomization (MR) [147]. Beyond genetic instruments, causal inference in longitudinal omics can also be approached using non-Mendelian frameworks, including time-varying instrumental variables, g-methods, structural equation models, and functional causal models. A quasi-experimental study [148] was designed using instrumental variables in the form of single-nucleotide polymorphisms as a way to estimate the causal effect of a biomarker of interest, usually through variations of two-stage linear models, potentially with random effects. One may use both MR and LMMs to estimate the causal effect of metabolite biomarkers in a longitudinal cohort [149], while another study used functional data analysis methods [150] to account for time-varying exposures when estimating the effect of biomarkers on outcomes of interest. These non-Mendelian approaches are particularly appealing in longitudinal settings, where repeated measurements enable modeling of time-dependent confounding and dynamic causal effects. Since MendelianRandomization and TwoSampleMR packages to implement MR currently focus on cross-sectional data, extending both Mendelian and non-Mendelian causal frameworks to longitudinal omics contexts remains an active area of research.

## 8 Discussion

We highlighted key challenges in LOD analysis, where classical methods such as LMM and GLMM face limitations related to intra-subject correlation, high-dimensionality, irregular sampling, and distributional assumptions. While mixed-effect models remain foundational, nonparametric solutions for complex correlation structures are still underdeveloped, and existing imputation strategies often fail to preserve biologically meaningful temporal variability. The comparative framework provided in Table 1 illustrates that no single method is uniformly optimal across longitudinal omics settings, underscoring the importance of aligning methodological choices with data characteristics and scientific objectives.

Multivariate methods are particularly limited when handling high-dimensional omics data, as most approaches fail beyond 10–20 response variables [110], [151]. The redundancy of certain genes at specific time points further complicates analysis. Nonparametric FDA methods offer potential solutions but remain computationally demanding. Additionally, log-transformation normality assumptions often fail in real datasets, and Bayesian extensions for LMM/GLMM in high-dimensional settings are lacking. Approaches such as joint modeling of survival and longitudinal data improve efficiency, reduce bias, and enable dynamic predictions. While many software tools exist, their scalability is uncertain, as most were developed for specific datasets and require regularization for broader applications. Future research should focus on computationally efficient and adaptable methods for high-dimensional LOD.

Computational scalability represents an additional practical constraint for many advanced longitudinal models. Gaussian process regression and Bayesian hierarchical formulations often require approximation strategies such as sparse kernels, low-rank representations, variational inference, or inducing-point methods to remain tractable. Parallelization across features or subjects is commonly used in practice, although it does not fully resolve memory or convergence limitations. More recently, GPU-accelerated inference and deep learning–based surrogates have been explored to scale longitudinal modeling to large omics datasets, but these approaches introduce new trade-offs in interpretability and reproducibility.

A major unresolved challenge concerns ultra-high-dimensional longitudinal features, particularly in transcriptomics and other sequencing-based assays. Although regularization approaches such as LASSO and elastic net are widely used, applying them directly to tens of thousands of longitudinal features can still lead to overfitting and unstable inference. This highlights the need for systematic investigation of feature pre-screening strategies, such as sure independence screening, correlation-based filtering, or stability-driven screening, prior to longitudinal modeling. Despite their use in cross-sectional settings, the impact of such screening procedures on bias, power, and temporal inference in longitudinal omics remains largely unexplored and represents an important methodological gap.

Longitudinal multiomics integration presents a related bottleneck. While numerous methods exist for cross-sectional multiomics integration, longitudinal settings remain dominated by either layer-wise modeling or early feature concatenation followed by joint analysis. This practice is driven largely by computational and identifiability constraints, as fully joint longitudinal models scale poorly with increasing numbers of omics layers, features, and time points. As a result, most current longitudinal multiomics analyses sacrifice temporal resolution or cross-layer structure to maintain tractability, limiting their ability to capture dynamic biological regulation.

Importantly, explicit cross-omics *interaction* modeling over time is still rare: most studies quantify cross-layer relationships through association analyses, but few directly estimate time-varying interaction effects within a unified longitudinal framework. Developing statistically principled models that can quantify dynamic interactions between molecular layers (e.g., transcriptome-metabolome or microbiome-host signaling) while accounting for repeated measures and imbalances remains an open challenge.

Finally, methodological development must be accompanied by context-specific benchmarking. The utility of longitudinal omics methods cannot be fully assessed by predictive accuracy alone; instead, evaluations should consider pathway recovery, temporal ordering, robustness to sampling irregularity, and biological interpretability within specific disease or experimental contexts. Dedicated benchmarking studies tailored to clinical domains such as autoimmune diseases, cancer, or neurodegeneration are needed to guide method selection and to assess whether models recover biologically plausible longitudinal mechanisms rather than statistical artifacts.

We summarized key methods for longitudinal omics analysis and noted available software tools, highlighting their strengths and limitations. Scalable and flexible models are still needed to handle high-dimensional, irregular, and sparse data. Developing statistically principled frameworks for explicit longitudinal cross-omics interaction modeling remains an important open direction. Combining nonparametric, Bayesian, and deep learning approaches will be key to future progress.

## CRediT authorship contribution statement

**Ali Reza Taheriyoun:** Writing – review & editing, Writing – original draft, Visualization, Validation, Software, Resources, Methodology, Investigation. **Allen Ross:** Writing – review & editing. **Abolfazl Safikhani:** Writing – review & editing. **Damoon Soudbakhsh:** Writing – review & editing, Conceptualization. **Ali Rahnavard:** Writing – review & editing, Writing – original draft, Visualization, Validation, Supervision, Project administration, Methodology, Funding acquisition, Conceptualization.

## Declaration of competing interest

The authors declare that they have no known competing interests or personal relationships that could have appeared to influence the work reported in this paper.
