# Supplementary material for: Longitudinal omics data analysis: approaches and applications
Source: Comput Struct Biotechnol J. 2026 Jan 5;31:301–15. doi: 10.1016/j.csbj.2026.01.001 (PMC12818117; doi:10.1016/j.csbj.2026.01.001)
Supplement: Multimedia Component 1 [file mmc1.pdf]

Supplementary Material for Longitudinal Omics Data Analysis: Approaches and Applications

Ali Reza Taheriyoun[1], Allen Ross[1], Abolfazl Safikhani[2], Damoon Soudbakhsh[3], Ali Rahnavard[1,*]

A. Abbreviations, summary table and algorithm to read the review

Table A.1: Alphabetically sorted list of abbreviations used in the manuscript.

Abbreviation	Full Form	Abbreviation	Full Form
AIC	Akaike Information Criterion	AIDS	Acquired Immunodeficiency Syndrome
AR	Autoregressive	AUC	Area Under the Curve
BGC	Biosynthetic Gene Cluster	B–H	Benjamini–Hochberg
BIC	Bayesian Information Criterion	BMI	Body Mass Index
BUGS	Bayesian Inference Using Gibbs Sampling	CALDER	Clonal Architecture Inference Using Longitudinal Data and Error Correction
CBC	Complete Blood Count	CD4	Cluster of Differentiation 4
CHAMACOS	Center for the Health Assessment of Mothers and Children of Salinas	CI	Confidence Interval
COVID-19	Coronavirus Disease 2019	DEC	Damped Exponential Correlation

Continued on next page

*Corresponding author rahnavard@gwu.edu

[1]Department of Biostatistics and Bioinformatics, The George Washington University,800 22nd St NW, Washington, 20052, DC, USA

[2]Department of Statistics, George Mason University, Fairfax, 22030, VA, USA

[3]Department of Mechanical Engineering, Temple University, Philadelphia, 19122, PA, USA

Abbreviation	Full Form	Abbreviation	Full Form
DEA	Differential Expression Analysis	DNA	Deoxyribonucleic Acid
EC	Estradiol Concentration	EEN	Exclusive Enteral Nutrition
EM	Expectation–Maximization	FDA	Functional Data Analysis
FDR	False Discovery Rate	FPC	Functional Principal Component
GEE	Generalized Estimating Equations	GLM	Generalized Linear Model
GLMM	Generalized Linear Mixed Model	GMM	Generalized Method of Moments
GP	Gaussian Process	GWAS	Genome-Wide Association Study
HCV	Hepatitis C Virus	HIV	Human Immunodeficiency Virus
iDREM	Integrative Dynamic Regulatory Events Miner	IBD	Inflammatory Bowel Disease
IBS	Irritable Bowel Syndrome	LACE	Longitudinal Analysis of Cancer Evolution
LDA	Linear Discriminant Analysis	LH	Luteinizing Hormone
LMM	Linear Mixed Model	LOD	Longitudinal Omics Data
LRT	Likelihood Ratio Test	MAPTest	Maximum Average Power Test
MAR	Missing at Random	MCAR	Missing Completely at Random
MCMC	Markov Chain Monte Carlo	MLE	Maximum Likelihood Estimator
MNAR	Missing Not at Random	mRNA	Messenger RNA
MSE	Mean Squared Error	MtLMM	Multivariate t-student Linear Mixed Model
miRNA	MicroRNA	NLMIXED	Nonlinear Mixed Effects procedure in SAS
NNRTI	Non-Nucleoside Reverse Transcriptase Inhibitor	OLS	Ordinary Least Squares

Continued on next page

Abbreviation	Full Form	Abbreviation	Full Form
PBMC	Peripheral Blood Mononuclear Cells	PCA	Principal Component Analysis
PG	Progesterone	PI	Protease Inhibitor
PSA	Prostate-Specific Antigen	QIFC	Quadratic Inference Function Classifier
REML	Restricted Maximum Likelihood	RF	Random Forest
RNA	Ribonucleic Acid	scATAC	Single-Cell Assay for Transposase-Accessible Chromatin
scRNA	Single-Cell RNA	SNP	Single Nucleotide Polymorphism
SWAN	Study of Women's Health Across the Nation	T1D	Type 1 Diabetes
TNF	Tumor Necrosis Factor	UMAP	Uniform Manifold Approximation and Projection
ZDV	Zidovudine		

B. Reproducible Examples, Code and Data Availability

To enhance the practical utility and reproducibility of this review, we provide fully reproducible implementations for several representative longitudinal omics analyses discussed in the manuscript. All scripts, simulation code, and example analyses are publicly available at http://github.com/omicsEye/LODReview. The repository contains the following worked examples, each corresponding to figures or methods presented in the main text:

- **Linear mixed-effects models (LMM) and generalized estimating equations (GEE):** Complete `R` scripts used to generate Fig.3 , illustrating the impact of balanced versus imbalanced sampling on longitudinal inference. These scripts reproduce the simulation study, model fitting, estimation of time effects, and visualization shown in the manuscript.

- **Bayesian and logistic classifiers for longitudinal data:** Reproducible code for Fig. 4, comparing Bayesian and logistic classification approaches under longitudinal designs. The scripts include data splitting, model training, and evaluation using misclassification rates.

- **Zero-inflated and compound Poisson mixed models for microbiome data:** An applied example using data from the MGTX clinical trial [1], demonstrating compound Poisson modeling for sparse longitudinal microbiome features. The repository includes both the modeling code and the associated input tables.

- **Functional and Gaussian processbased longitudinal modeling:** An illustrative example using the `waveome` framework for functional data analysis of longitudinal CD4 count data, demonstrating nonparametric modeling of irregularly sampled trajectories.

These examples are intended to serve as minimal, practical templates that readers can adapt to their own longitudinal omics studies.

The `Categorized References.csv` file summarizes the entry documents. The 0/1 values indicate whether an article addresses or whether the mentioned methodology *can* address the subject listed in each column title. The last three columns specify whether the proposed methodology in the entry document *can* potentially be used for genomic data, differential expression analysis (DEA), or time-course data. Algorithms A1 and A2 help break down the study's scientific question into general components and point to the relevant sections of the manuscript that address each one.

Algorithm A1: Overall steps in the analysis of LOD

```
Data: Omics and metadata tables
if number of omics features of interest < 10 [2] then
    if multivariate normal or t distribution assumption is fulfilled then
        Choose one of the methods discussed in the "Multivariate Outcomes" Section, considering the balance of the sampling
          design.
    else
        implement algorithm A2
    end
else
    if the question is "Are the omics features affected by the metadata?" then
        implement algorithm A2
    else
        if The scale of the metadata of interest is count or continuous then
            if normality assumption is fulfilled for the biomarker measurements then
                Use high-dimensional LMM with or without survival analysis (depending on the study design), considering
                  the balance of the sampling design.
            else
                Use the corresponding high-dimensional GLMM depending on the distribution of biomarker measurements,
                  with or without survival analysis (depending on the study design), considering the balance of the sampling
                  design.
            end
        else
            Depending on the sampling design balance, use one of the methods reviewed in "Classification based on LOD"
              Section, or apply joint modeling of GLMM across survival as discussed in Appendix H.
        end
    end
end
```

Algorithm A2: Univariate FDR

Data: Omics and metadata tables
for *each omics feature* **do**
  if *normality assumption is fulfilled* **then**
    Consider the sparsity of data and balance of sampling design;
    if *data is not survival* **then**
      fit one of the LMM based methods discussed in "Linear mixed models" SubSection;
    else
      fit one of the joint models in Appendix H;
    end
  else
    if *there exists a non-Gaussian underlying distribution for the feature with particular link function which determines the relationship between the average value of omics feature and 'linear' function of metadata* **then**
      fit one of the GLMM-based methods discussed in "Abundances and non-Gaussian relative abundances" SubSection;
    else
      estimate unknown relationship between the average value of omics and metadata, 'nonparametrically';
      compute the significance of each covariate using methods [3];
    end
  end
  save the corresponding p-values for the corresponding coefficients;
end
compute adjusted p-value using methods like B-H;
threshold the adjusted p-values;
determine significantly associated features and variables;

C. Linear and generalized linear mixed effects models

Let $\mathbf{y}_i$ be the $n_i \times 1$ vector of all measured values (such as count, abundance, relative abundance, intensity, etc.) from the ith subject (patient) for $i = 1, \ldots, m$. The metadata provides the $n_i \times p$ design matrix of the fixed effect of ith subject denoted by $\mathbf{X}_i$. Gene expressions and microbiome information of each subject, like a fingerprint, are uniquely dedicated to that subject, and thus, multiple measurements from the same subject produce intra-subject correlation, which must be included in the models. The dependence structure of the multiple measurements out of an individual is explained by unknown individual effect, $\mathbf{b}_i$ with known $n_i \times k$ design matrix $\mathbf{Z}_i$ which links the unknown *random effect* $\mathbf{b}_i$ to the observed measurements $\mathbf{y}_i$ by

$$\mathbf{y}_i = \mathbf{X}_i\boldsymbol{\beta} + \mathbf{Z}_i\mathbf{b}_i + \boldsymbol{\varepsilon}_i, \tag{S.1}$$

for $i = 1, \ldots, m$ where $\boldsymbol{\varepsilon}_i \overset{iid}{\sim} N(\mathbf{0}, \boldsymbol{\Sigma}_{\text{Err}})$ is Gaussian white noise independent of the Gaussian random effects $\mathbf{b}_i \overset{iid}{\sim} N(\mathbf{0}, \boldsymbol{\Sigma}_{\text{rndEff}})$ and in this way

$$\text{Var}(\mathbf{y}_i) = \boldsymbol{\Sigma}_{\text{Err}} + \mathbf{Z}_i\boldsymbol{\Sigma}_{\text{rndEff}}\mathbf{Z}_i^{\top}, \tag{S.2}$$

which reflects the intra-subject dependence in the second term. Eliminating this correlation from the analysis is equivalent to treating the random effect as a constant effect ($\Sigma_{\text{rndEff}} = \mathbf{0}$), which leads to incorrect inference about $\boldsymbol{\beta}$ and underestimates the variance of the estimates. In the imbalanced case, the time effect is often incorporated into $\mathbf{Z}$; that is, its columns represent the multiplication of time or the squared value of time by the fixed effects. This leads to a nonstationary covariance structure for $\mathbf{y}_i$ even within a subject, meaning that the trajectory $y_{i1}, \ldots, y_{in_i}$ observed as responses of subject i at times $t_{i1}, \ldots, t_{in_i}$, is a realization of a nonstationary process, regardless of the mean function. A better understanding of this analysis can be gained from a two-stage modeling approach: first, fitting a linear model between each subject's measurements and the time-related components (intercept, time, square value of time, etc.), and then regressing the m groups of resulting coefficients onto the fixed effects. The maximum likelihood estimator for the LMM described above has a considerable computational cost. Therefore, an efficient EM algorithm [4] is usually hired to compute the REML estimates.

Gaussianity is a very restrictive assumption for abundance count data, necessitating a generalization of the LMM. The GLMM [5] parameterizes the conditional expectation of the response variable as

$$E[\mathbf{y}_i|\mathbf{b}_i] = g^{-1}(\mathbf{X}_i\boldsymbol{\beta} + \mathbf{Z}_i\mathbf{b}_i), \tag{S.3}$$

where $g^{-1}(\cdot)$ is the inverse link function and $\mathbf{Y}|\mathbf{b}$ belongs to the exponential dispersion family. Despite the flexibility and covering a wide spectrum of distributions, including count and ordinal data, this model has often not been applied to the analysis of LOD. Instead, researchers frequently either ignore the proper likelihood assumptions or use a concave transformation, such as $\log(\cdot)$, to use the normal approximation. Computing the GLMM estimates, whether using frequentist or Bayesian approaches, remains challenging. Researchers interested in using GLMM for their longitudinal studies can employ `glmm` [6], `glmmML` (for Poisson and binomial responses) `R` packages for univariate continuous or discrete response data and `glmmTMB` [7] for zero-inflated features. For multivariate analysis, `MCMCglmm` [8] and `brms` [9] are recommended, with `brms` specifically considering the survival data. The last two packages are implemented within a Bayesian statistical framework.

Model (2) is also used to address a different class of models involving time-varying covariates. Here, we incorporate the effect of time as a ratio scale to highlight the dependency of the covariate $\mathbf{X}^\top = (\mathbf{X}_{it_{i1}}| \ldots |\mathbf{X}_{it_{in_i}})$ where $\mathbf{X}_{it_{ij}}^\top$ is the row vector of covariates for the ith subject at time t_{ij}. Note that the covariates are not only random but also constitute a stochastic process. This aspect is often overlooked in the analysis of LOD, where metadata are typically treated as fixed effects without accounting for their random structure. While this approach does not introduce additional bias into the ordinary least squares (OLS) estimates of the parameters, it does affect the significance of test results. According to the basic types of time-varying covariates [10], two well-known types of modeling are subject-specific and population-averaged models. The subject-specific model is a specific version of the GLMM, considering the subject effect as one of the random effects given by

$$y_{it_{ij}}|s_i \sim \mathcal{L}(\mu(\mathbf{X}_{it_{ij}}, \mathbf{Z}_i)), \quad \text{where} \quad s_i \overset{iid}{\sim} F_s,$$

where $\mathcal{L}$ denotes the distribution of response with mean $\mu(\mathbf{X}_{it_{ij}}, \mathbf{Z}_i)$ and F_s is the distribution of the random subject effect s_i. The systematic part is formulated using a link function as in (2). In population-averaged models, the

response variable is linked to the subject's time-varying behavior via the mean function rather than the process $\{\mathbf{x}_{it}\}_t$. Specifically,

$$y_{it_{ij}}|s_i \sim \mathcal{L}(\mu(\mathbf{X}_{it_{ij}}), \phi V(\mu(\mathbf{X}_{it_{ij}}))),$$

where $V(\cdot)$ is the variance function when the process $\{\mathbf{x}_{it}\}_t$ is nonstationary, and the distribution $\mathcal{L}$ belongs to exponential dispersion family. The conditional mean of the distribution is linked to the covariates and random effects. The generalized estimating equations (GEE) available via `R` package `geepack` [11] or generalized method of moments (GMM) are commonly used for parameter estimation in time-dependent models.

We bring the use of LMMs or GLMMs with time-varying factors in longitudinal omics studies into attention because these models typically assume that covariates have been fixed (baseline values). When the value of a feature in the metadata depends on its previous value or outcome at an earlier time point, it becomes an endogenous factor, which can cause bias in estimating metadata effects. This important and widespread issue was discussed by Qi et al. [12] in the literature and further elaborated in a rejoinder version [13]. They proposed a mechanism that, under a conditional independence assumption, allows the use of standard software for fitting LMMs in the presence of endogenous factors. Without this approach, studies would need to develop their methodologies to control for the bias introduced by endogenous factors.

D. Supplementary figures

E. DEA from hypothesis testing viewpoint

DEA in longitudinal studies aims to identify features that change across conditions and/or over time. Let $\mathbf{y}_{ig}$ be the vector of all read counts for an omics feature (such as a gene) g from subject i, and suppose that $\mathbf{y}_{ig} \sim p(\cdot|\boldsymbol{\theta}_g, \boldsymbol{\eta}_g)$ where $\boldsymbol{\theta}_g$ is the parameter vector that captures changes over time and $\boldsymbol{\eta}_g$ represents nuisance parameters. GLMM can be useful for incorporating the random effects of subjects by replacing $\boldsymbol{\theta}_g$ with $\boldsymbol{\theta}_{ig}$. The primary focus in DEA is hypothesis testing on $\boldsymbol{\theta}$ rather than estimation or prediction. In this context, hypothesis testing involves simultaneous testing, where concepts such as the false discovery rate (FDR) or the expected false positive rate become more relevant than the traditional type I error. If the hypotheses take the form $H_0^g : \boldsymbol{\theta}_g \in \Theta_0$ versus $H_1^g : \boldsymbol{\theta}_g \in \Theta_1$, a widely used approach is multiple Bayesian testing based on the Bayes factor

$$\delta(\mathbf{y}) = \frac{Pr(\boldsymbol{\theta}_g \in \Theta_0) \times Pr(\mathbf{y} \in \mathcal{R})}{Pr(\boldsymbol{\theta}_g \in \Theta_1) \times Pr(\mathbf{y} \notin \mathcal{R})}, \tag{S.1}$$

where $\mathcal{R} = \{\mathbf{y} : (\sum_{g=1}^{G_0} p(\mathbf{y}|\boldsymbol{\theta}_g, \boldsymbol{\eta}_g))(\sum_{g=G_0+1}^{G} p(\mathbf{y}|\boldsymbol{\theta}_g, \boldsymbol{\eta}_g))^{-1} \leq s\}$, is the rejection region, G_0 is the number of true nulls and G is the total number of genes. For instance, consider the case of two time points, t_1 and t_2 and two conditions c_1 and c_2. Suppose $\boldsymbol{\theta}_g$ is of the form $(\theta_{g11}, \theta_{g12}, \theta_{g21}, \theta_{g22})$, where θ_{gct} represents the mean of the distribution of the omics feature under condition c at time point t. Then, $H_0^g : \boldsymbol{\theta}_g \in \Theta_0 = \{\boldsymbol{\theta}_g : \theta_{g11} + \theta_{g12} = \theta_{g21} + \theta_{g22}\}$ tests the effect of condition, while $H_0^g : \boldsymbol{\theta}_g \in \Theta_0 = \{\boldsymbol{\theta}_g : \theta_{g11} + \theta_{g21} = \theta_{g12} + \theta_{g22}\}$ tests the effect of time. For large enough G_0, G, and $G - G_0$, the Bayes factor does not depend on G_0. Hwang and Liu [42] demonstrated

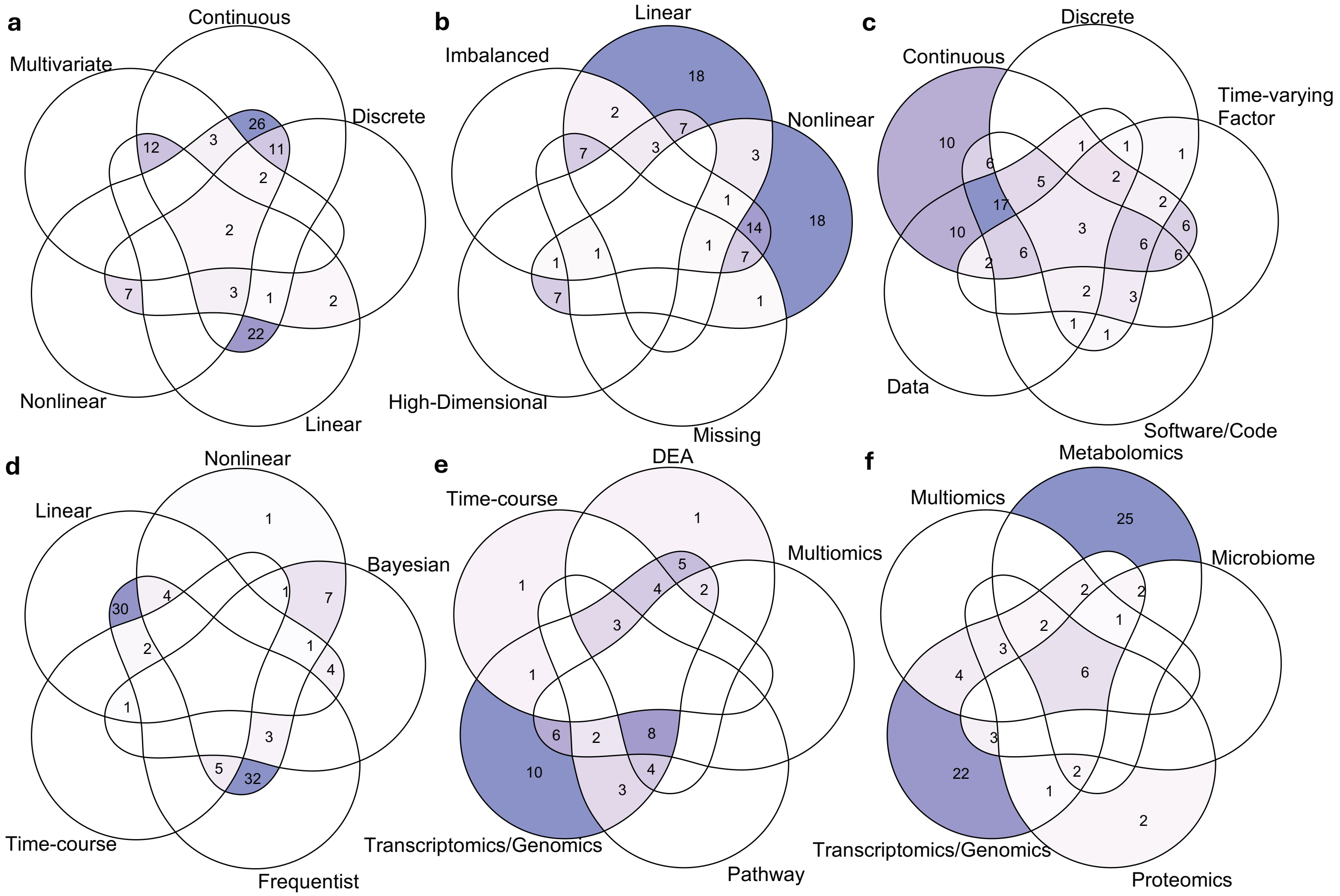

Fig. S1: The number of each study with LOD we have considered from different points of view. **a** shows how studies cover the analyses by dealing with the abundances as discrete or continuous variables. It also differentiates between the methodologies that use multivariate approaches rather than the ones that use multiple univariate methods. **b** demonstrates the same idea from the sample design viewpoint and categorizes the references if they have considered the effect of imbalanced sample designs and missing values. **c** shows the availability of the computational tools or codes as well as the publicly available data across the variable types (count/relative abundance) by categorizing the fixed effects as time-varying or observed only at the baseline. **d** shows the frequencies of approaches used by the studies from Bayesian or frequentist viewpoints. It also shows the frequency of time-course studies. **e** categorizes the studies from the type of biological approach. **f** demonstrates the distribution of studies across the omics types by considering the multiomics studies.

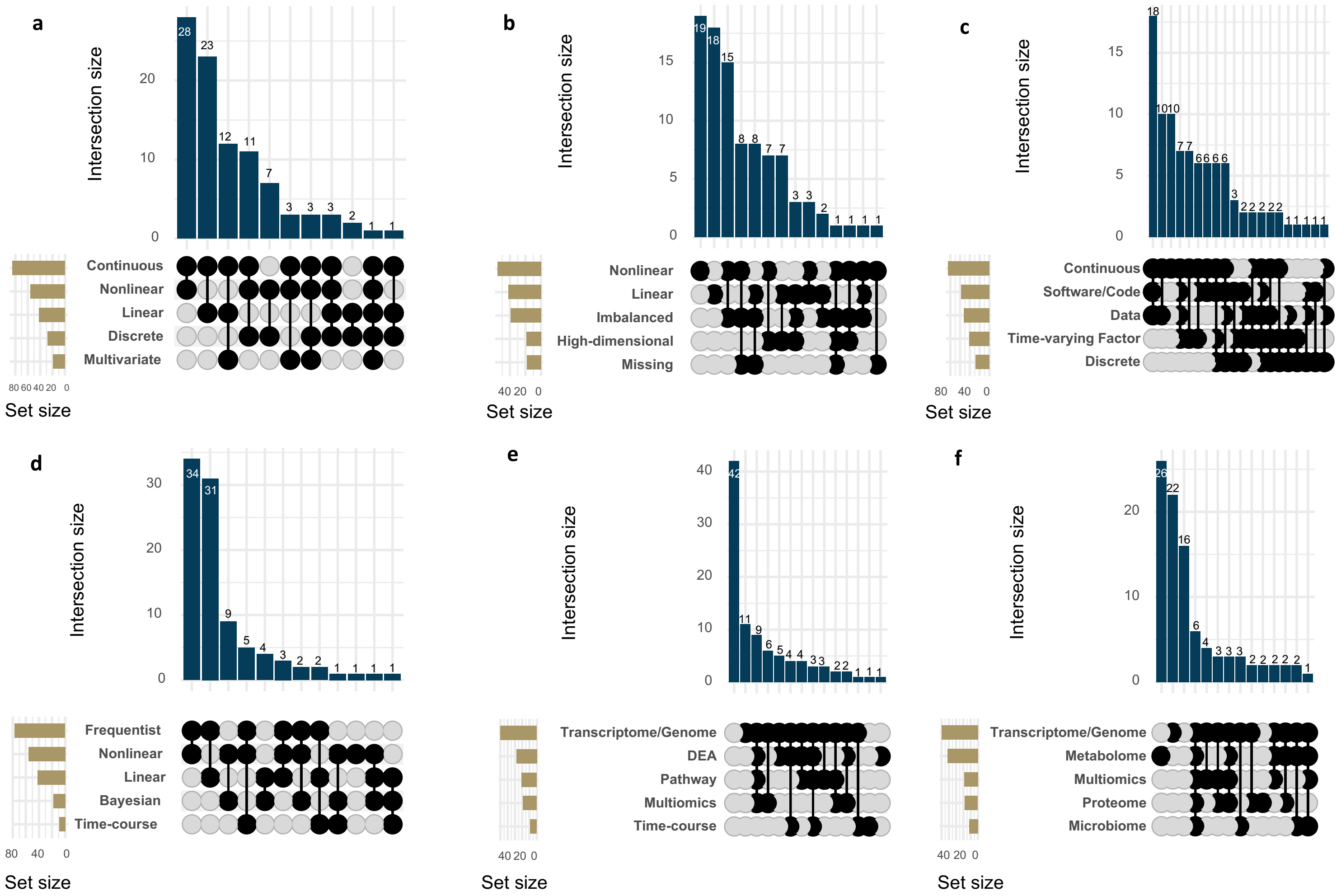

Fig. S2: The upset plot for the coverage of reviewed references from different points of view. Each column represents a unique intersection of methodological categories, indicated by connected black dots in the matrix below; bar heights denote the number of studies in each intersection. **a** shows how studies cover the analyses by dealing with the abundances as discrete or continuous variables. It also differentiates between the methodologies that use multivariate approaches rather than the ones that use multiple univariate methods. **b** demonstrates the same idea from the sample design viewpoint and categorizes the references if they have considered the effect of imbalanced sample designs and missing values. **c** shows the availability of the computational tools or codes as well as the publicly available data across the variable types (count/relative abundance) by categorizing the fixed effects as time-varying or observed only at the baseline. **d** shows the frequencies of approaches used by the studies from Bayesian or frequentist viewpoints. It also shows the frequency of time-course studies. **e** categorizes the studies from the type of biological approach. **f** demonstrates the distribution of studies across the omics types by considering the multiomics studies.

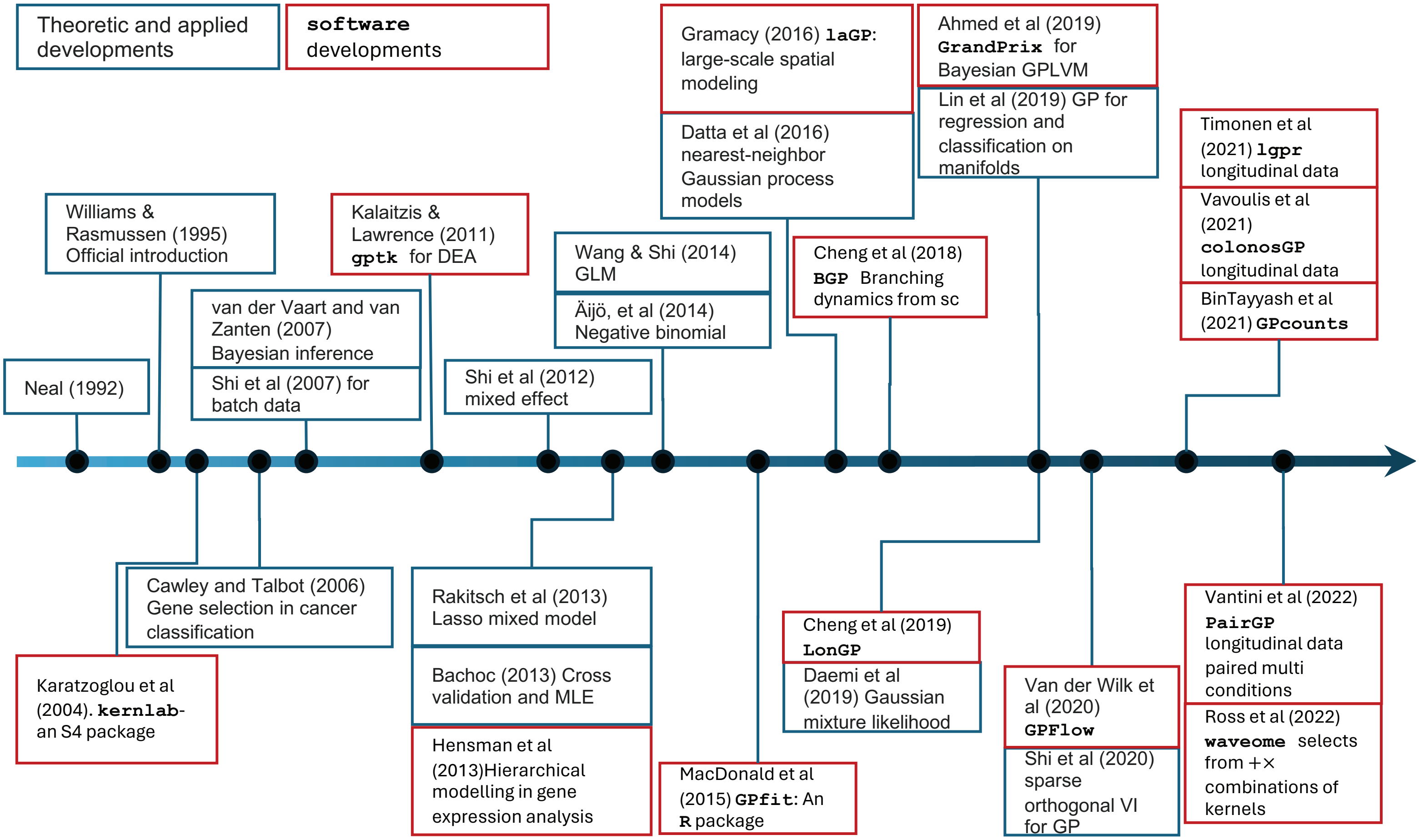

Fig. S3: The evolution of GP approach across the time [14–41].

that the value of s can be calculated to keep the FDR below a nominal level, and based on the Neyman-Pearson lemma, it maximizes the average power.

One straightforward method in DEA for LOD is to fit an LMM or GLMM, and then apply an appropriate series of tests with the FDR correction to properly implement simultaneous testing. For example, returning to the GLMM (3) and (4), after computing the maximum likelihood estimates (MLE) of the parameters, likelihood ratio tests (LRTs) are also available to assess the effect of fixed effects on the presence of bacterial taxa ($H_0 : \boldsymbol{\alpha} = \mathbf{0}$). The association of taxa with covariates can also be identified using the LRT for ($H_0 : \boldsymbol{\beta} = \mathbf{0}$). This method can be used with any GLMM to correctly account for the effect of intra-subject correlation. The estimation and testing are available in `R` package `ZIBR`.

F. LonDA

Linear discriminant analysis (LDA) is also a widely used method in omics data analysis. The main aim of a classification problem is to prediction of the label of new samples based on the provided data. This problem becomes more complicated by adding the time effect in longitudinal data. Moreover, in an omics study and particularly in rare disease studies the number of subjects labeled 'case' is severely outnumbered by 'control' subjects, which tends to imbalanced samples. A methodology for the longitudinal discriminant analysis (LonDA) was introduced by Marshall and Barón [43]. Simply speaking, LonDA classifies the samples into predefined labels based on the observed LOD of each sample. In practice, each subject at each time point is labeled, and the LonDA provides a model based on the observed training set of longitudinal data to predict the label of unlabeled subjects. In more detail, LonDA is a classification model that considers the change in the parameter set of the distribution of each group across time. Let G_j, $j = 1, \ldots, g$ denotes g labels (populations) and $\mathbf{y}_i$ be the vector of observed values (data) for subject i on arbitrary times $\mathbf{t} = (t_1, \ldots, t_{n_i})^t op$ then the corresponding distribution of $\mathbf{y}_i$ would be $f_j(\mathbf{y}_i; \phi_j(\mathbf{t}))$ where the dimension of density parameters, $\phi_j(\mathbf{t})$, depends on the number of replicates (or dimension of $\mathbf{t}$) per each subject. For prior probabilities $\pi_1, \ldots, \pi_g$, the decision boundary [44] is

$$\log \pi_k + \log f_k(\mathbf{y}_i; \phi_k(\mathbf{t})) = \max_j \{\log \pi_j + \log f_j(\mathbf{y}_i; \phi_j(\mathbf{t}))\}, \qquad j = 1, \ldots, g. \tag{S.1}$$

If the densities are Gaussian with the same covariance matrices, the decision boundaries are linear. Even in this case, the mean value of the jth population, $\boldsymbol{\mu}_j(\mathbf{t}; \boldsymbol{\alpha}_j)$ say, can make a nonlinear relationship with the covariates where $\boldsymbol{\alpha}$ is the mean population specific parameter. The rest of the modeling procedure returns to the estimation of the parameters. For instance, if $\mathbf{V}_j(\mathbf{t}, \boldsymbol{\alpha}_j; \boldsymbol{\theta}_j)$ be the variance matrix of vector $\mathbf{y}$ in the population j, where $\boldsymbol{\theta}_j$ is the variance specific parameter of population j, then regarding (S.2) one can write

$$\mathbf{V}_j(\mathbf{t}, \boldsymbol{\alpha}_j; \boldsymbol{\theta}_j) = \mathbf{Z}(\mathbf{t}; \boldsymbol{\alpha}_j)\boldsymbol{\Sigma}_{\text{rndEff}}(\boldsymbol{\theta}_j)\mathbf{Z}^\top(\mathbf{t}; \boldsymbol{\alpha}_j) + \boldsymbol{\Sigma}_{\text{Err}}(\boldsymbol{\theta}_j),$$

where $\mathbf{Z}(\mathbf{t}; \boldsymbol{\alpha}_j)$ is the design matrix corresponding to the random effect in population j. Parameter vectors $\boldsymbol{\alpha}_j$ and $\boldsymbol{\theta}_j$ can be either identical with the mean and variance elements or link them to the covariates. Depends on the homogeneity of $\mathbf{Z}(\mathbf{t}; \boldsymbol{\alpha}_j)$ and the variance related parameters, $\boldsymbol{\theta}_j$, four estimation situations were expected [43]:

1. Homoscedastic Model: $\mathbf{Z}(\mathbf{t};\boldsymbol{\alpha}_j) = \mathbf{Z}(\mathbf{t})$ and $\boldsymbol{\theta}_j = \boldsymbol{\theta}$,
2. Mean-heteroscedastic model: $\mathbf{Z}(\mathbf{t};\boldsymbol{\alpha}_j)$ varies in j but $\boldsymbol{\theta}_j = \boldsymbol{\theta}$,
3. Variance-heteroscedastic model: $\mathbf{Z}(\mathbf{t};\boldsymbol{\alpha}_j) = \mathbf{Z}(\mathbf{t})$ but $\boldsymbol{\theta}_j$ varies in j, and
4. Fully-heteroscedastic model: Both the $\mathbf{Z}(\mathbf{t};\boldsymbol{\alpha}_j)$ and $\boldsymbol{\theta}_j$ vary in j.

The methodology was used in studying the subunit beta measurements of 174 pregnant women over 2 years in a private obstetrics clinic in Santiago, Chile [43]. The data were imbalanced since there was only one measurement for 30% of subjects, two for 31%, three for 33%, and 6% with more than three measurements. The subunit beta is used as a biomarker in this study to discriminate the samples with normal/abnormal delivery. Prior probabilities were the proportion of each group in the sample. The response variable was log subunit beta measurements and the response of the ith subject at time t_{ik} when it belongs to i^{th} group in the sample, $y^{(j)}_{i,t_k}$ for instance under the Mean-heteroscedastic model follows

$$y^{(j)}_{i,t_k} = \frac{\alpha_{j1} + b_{ij}}{1 + \alpha_{j2} e^{-\alpha_{j3} t_{ik}}} = \varepsilon_{i t_{ik} j},$$

where here, $i = 1, \ldots, 174$, $j = 1, 2$ and $t_i k$ is the kth time of measurement for subject i where $k = 1, \ldots, n_i$. In this study, $n_i = 1, 2, 3, > 3$ and suggests the notation $\boldsymbol{\alpha}_j = (\alpha_{1j}, \alpha_{2j}, \alpha_{3j})$. The remaining three models can be written in the same way and the fitted models chose the fully heteroscedastic model based on the deviance test, which confirms the longitudinal effects in both the mean and variance of the log subunit beta measurements in two groups. Although there is no restriction of using the same method on the multivariate case, the bivariate case of this model was specifically discussed [45] with considering the missing completely at random structure applied on the Estradiol and β-HCG concentrations but using the 161 subjects and it is mentioned that they employed `NLMIXED` procedure from `SAS` to estimate the parameters. The same data same model under fully-heteroscedasticity assumption using the nonlinear hierarchical classification were studied in a fully Bayesian approach [46]. A clustering method was also presented [47] based on a mixture decomposition scheme [48] and then performed the same classification problem under both the balanced and imbalanced cases for multivariate longitudinal data.

G. Bayesian approach on LMM and GLMM

Employing the Bayesian approach in longitudinal data analysis mostly happens through two main ideas: (1) A Bayesian statistical model (see Robert [49] Page 9 for the definition of the parametric version which considers a prior distribution on the parameters of a parametric statistical model) and (2) Bayesian learning as a machine learning paradigm by considering parameters as a latent variable [50]. Using either approach, the main step is a posteriori computation and mostly arises when the sample size is not large enough to achieve the usual asymptotics of LMM and GLMM from the frequentist's viewpoint. The main step in the Bayesian framework involves incorporating the expert's opinion into the prior distribution in such a way that updating the posterior information via data does not result in unmanageable computational complexity. Here, we only restrict our review to the Bayesian practice of LMM and GLMM. In the sequel, we are listing the names of some of the approaches covered by computational tools with an application on LOD for each that exists.

The problem of estimating the parameters of LMM in the Bayesian approach has been widely studied and performed in different software. We encourage the readers to consider the random effect selection in a hierarchical Bayesian framework [51], which also gives the idea of Bayesian modeling of LMM. `brms` [52] introduced a fully Bayesian framework for the GLMMs with the possibility of zero-inflation and also provided theoretically defined identifiable models. A very practical version of this approach, only for GLMM with restricted distributions, is conserved in `blme` [53]. `JMbayes` [54] is the Bayesian version of the joint modeling of LMM and survival models `JM` [55]. `APML0` [56] has been designed for longitudinal genome data and considers the phenotype as the response variable and the genome as a fixed effect. It provided a fast $\mathbb{L}_0$-penalized estimator for the genes effect and nominated the important genes as biomarkers. Bayesian model selection by considering the marginal and conditional criteria for count data is available in `BayesselectGLMM` function in `R` [57] which provides a computationally adaptive method to compute the Bayes and pseudo-Bayes factors. The method was examined on clinical data; however, in genome-wide data, where usually each gene is modelled versus metadata, the same approach is applicable on the abundance table. The `Stan` stand-alone software [58] also can be hired for Bayesian estimation of model (1) per each response variable. There is also a guideline to use `winBUGS` package in `R` for the general framework of Bayesian GLMM, which can be used for longitudinal data. `bmrarm` is designed to jointly model the longitudinal continuous and ordinal responses in the Bayesian domain [59]. This can help mapping the longitudinal metadata onto baseline omics features. Some longitudinal omics studies, although they did not employ a Bayesian analysis, used the normalized counts generated by `DESeq2` [60], which is essentially a Bayesian GLM estimation tool under a negative binomial distribution. This approach can be found for single-cell data [61] and in previously discussed transcriptome data [62].

H. Survival, dropout and sparsity

In omics studies, particularly for severe diseases, subjects may leave before the study ends due to treatment changes, withdrawal, recovery, or death. These scenarios require specific modeling, often incorporating the time of exit. When the study focuses on this time (e.g., recovery/mortality), survival models are used. A key challenge is predicting disease outcomes based on LOD, as survival data is often sparse, with each subject measured only once. However, some studies integrate survival information with longitudinal measurements to model disease progression and predict survival. Notable examples include survival prediction using CD4 counts in AIDS cohorts [63, 64] and PSA levels in prostate cancer [65]. While survival datasets record time-to-event, single-time measurements are not considered longitudinal. This review focuses on survival studies with multiple time point measurements.

Most longitudinal omics studies employ Cox regression for survival analysis, focusing on time-to-event data [62]. While many use Cox models independently of omics data, some integrate joint modeling to assess the impact of longitudinal omics and baseline metadata, or baseline omics and longitudinal clinical data, on hazard rates.

H.1. Joint Linear Mixed and Cox Regression Models

In longitudinal treatment studies, joint models assess repeated measures while linking covariate effects (e.g., treatments) to time-to-event outcomes, such as biomarker level-crossing or dropout. A classic example is Zidovudine

(ZDV) treatment for HIV, where CD4 counts serve as surrogate markers for survival [63]. A double-blind placebo-controlled trial with 281 advanced HIV patients (144 receiving $250mg$ ZDV every four hours and the rest receiving placebo) measured CD4 levels periodically. The survival time T^* was modeled using CD4 levels (y_{ij}) and treatment within an LMM (1). With right-censoring at $T = \min\{T^*, C\}$, Cox regression estimated the hazard function $\lambda(t|\mathbf{y}) = \lambda_0(t)g(\mathbf{y}, \boldsymbol{\gamma})$. Nonrandom missingness introduced bias, as lower CD4 levels increased dropout probability. The random effect (time trend) was estimated via empirical Bayes [4] adjusted for missing. The `brms` package in `R` estimates model parameters without missing data adjustments [63]. Later, an EM algorithm was introduced for joint LMM-Cox regression estimation, yielding comparable results [66]. The extended change-point model is discussed in the next section.

The `JSM` package [67] jointly models longitudinal data using LMM and transformation models for survival analysis. The random effect function is estimated nonparametrically, and the baseline hazard function is modeled semiparametrically. Biomarkers serve as response variables, incorporating baseline data as fixed effects.

H.2. Integrated Ornstein-Uhlenbeck Random Effect

The two-stage joint model [63] was extended to include time-varying coefficients in CD4 studies. Data from 115 seroconverters estimated the infection time as the median between the last negative and first positive HIV test, excluding early CD4 measurements due to abrupt changes. Disease progression or last visit was modeled as:

$$\begin{cases} y_{ij} &= \psi_i(t_{ij}) + \varepsilon_{ij}, \\ \psi_i(t) &= a_i + bt + \mathbf{X}_i\boldsymbol{\beta} + W_i(t), \end{cases}$$

where a_i is the random intercept, and $W_i(t)$ follows an Ornstein-Uhlenbeck process. The hazard function $\lambda(t|\phi_i(t), \mathbf{X}^*) =$ $\lambda_0 \exp(\gamma\psi_i(t) + \mathbf{X}^*\boldsymbol{\beta}^*)$ was estimated via Bayesian approach and MCMC [64]. The dataset was highly imbalanced (132 visits per subject). Adjusting for ψ_i, baseline $\sqrt[4]{\text{CD4}}$, and age had minimal effects in the Cox model. Results confirmed declining CD4 counts negatively impacted survival ($b, \gamma < 0$), with reduced MSE compared to random effect models.

H.3. Change-Point Detection Model

Plasma HIV RNA data from the AIDS Clinical Trials Group 398 study [68] assessed virologic failure after 24 weeks in 481 patients across 12 time points over 72 weeks, comparing single and dual protease inhibitor (PI) regimens. Dropout and missing data led to an imbalanced design. Viral load trajectories were modeled as:

$$y_{ij} = \mathbf{X}_i\boldsymbol{\beta} + b_{i0} + b_{i1}t_{ij} + \sum_{l=1}^{L} \alpha_{il}(t_{ij} - \tau_l)_+ + \varepsilon_{ij} \coloneqq \psi_i(t_{ij}) + \varepsilon_{ij},$$

where the random effect design matrix is linear in time, with piecewise behavior at L change-points [69]. The error term follows $\varepsilon_{ij} \sim N(0, \sigma^2/\eta_i)$, allowing subject-specific variability.

Dropout was modeled using a Cox proportional hazards model $\lambda(t|\psi_i) = \lambda_0(t)\exp(\gamma\psi(t))$, where larger γ then a higher dropout rate. Parameters were estimated using a Bayesian MCMC approach, assuming missing-at-random data. Covariates included treatment (four arms: three dual-PI, one placebo-PI) and non-nucleoside reverse

transcriptase inhibitor (NNRTI). Results captured a significant NNRTI effect and a positive association between dropout and RNA viral load.

References

[1] Ali Reza Taheriyoun, Linda L. Kusner, Caitlin Loeffler, et al. Longitudinal transcriptomic analysis reveals signatures of treatment-response and thymectomy impact in myasthenia gravis. *submitted to the Journal of Clinical Investigations*, 2026.

[2] Geert Verbeke, Steffen Fieuws, Geert Molenberghs, and Marie Davidian. The analysis of multivariate longitudinal data: a review. *Stat. Methods Med. Res.*, 23(1):42–59, 2014.

[3] Pascal Lavergne, Samuel Maistre, and Valentin Patilea. A significance test for covariates in nonparametric regression. *Electronic Journal of Statistics*, 9(1):643 – 678, 2015.

[4] Nan M. Laird and James H. Ware. Random-effects models for longitudinal data. *Biometrics*, 38(4):963–974, 1982.

[5] N. E. Breslow and D. G. Clayton. Approximate inference in generalized linear mixed models. *Journal of the American Statistical Association*, 88(421):9, March 1993.

[6] Christina Knudson, Sydney Benson, Charles Geyer, and Galin Jones. Likelihood-based inference for generalized linear mixed models: inference with the `R` package `glmm`. *Stat*, 10:Paper No. e339, 9, 2021.

[7] Mollie E. Brooks, Kasper Kristensen, Koen J. van Benthem, et al. glmmTMB balances speed and flexibility among packages for zero-inflated generalized linear mixed modeling. *The R Journal*, 9(2):378–400, 2017.

[8] Jarrod D. Hadfield. MCMC methods for multi-response generalized linear mixed models: The MCMCglmm R package. *Journal of Statistical Software*, 33(2):1–22, 2010.

[9] Paul-Christian Bürkner. brms: An R package for Bayesian multilevel models using Stan. *Journal of Statistical Software*, 80(1):1–28, 2017.

[10] Tze Leung Lai and Dylan Small. Marginal regression analysis of longitudinal data with time-dependent covariates: a generalized method-of-moments approach. *J. R. Stat. Soc. Ser. B Stat. Methodol.*, 69(1):79–99, 2007.

[11] Søren Højsgaard, Ulrich Halekoh, and Jun Yan. The R package geepack for generalized estimating equations. *Journal of Statistical Software*, 15(2):111, 2005.

[12] Tianchen Qian, Predrag Klasnja, and Susan A. Murphy. Linear Mixed Models with Endogenous Covariates: Modeling Sequential Treatment Effects with Application to a Mobile Health Study. *Statistical Science*, 35(3):375 – 390, 2020.

[13] Tianchen Qian, Predrag Klasnja, and Susan A. Murphy. Rejoinder: Linear Mixed Models with Endogenous Covariates: Modeling Sequential Treatment Effects with Application to a Mobile Health Study. *Statistical Science*, 35(3):400 – 403, 2020.

[14] Radford Neal. Bayesian learning via stochastic dynamics. In S. Hanson, J. Cowan, and C. Giles, editors, *Advances in Neural Information Processing Systems*, volume 5. Morgan-Kaufmann, 1992.

[15] Christopher Williams and Carl Rasmussen. Gaussian processes for regression. In D. Touretzky, M.C. Mozer, and M. Hasselmo, editors, *Advances in Neural Information Processing Systems*, volume 8. MIT Press, 1995.

[16] Alexandros Karatzoglou, Alexandros Smola, Kurt Hornik, and Achim Zeileis. kernlab - an s4 package for kernel methods in r. *Journal of Statistical Software*, 11(9):120, 2004.

[17] Gavin C. Cawley and Nicola L. C. Talbot. Gene selection in cancer classification using sparse logistic regression with bayesian regularization. *Bioinformatics*, 22(19):2348–2355, 2006.

[18] Aad van der Vaart and Harry van Zanten. Bayesian inference with rescaled Gaussian process priors. *Electron. J. Stat.*, 1:433–448, 2007.

[19] J. Q. Shi, B. Wang, R. Murray-Smith, and D. M. Titterington. Gaussian process functional regression modeling for batch data. *Biometrics*, 63(3):714–723, 2007.

[20] Alfredo A Kalaitzis and Neil D Lawrence. A simple approach to ranking differentially expressed gene expression time courses through gaussian process regression. *BMC Bioinformatics*, 12(1), May 2011.

[21] J. Q. Shi, B. Wang, E. J. Will, and R. M. West. Mixed-effects Gaussian process functional regression models with application to dose-response curve prediction. *Stat. Med.*, 31(26):3165–3177, 2012.

[22] Barbara Rakitsch, Christoph Lippert, Oliver Stegle, and Karsten Borgwardt. A lasso multi-marker mixed model for association mapping with population structure correction. *Bioinformatics*, 29(2):206–214, 11 2012.

[23] François Bachoc. Cross validation and maximum likelihood estimations of hyper-parameters of Gaussian processes with model misspecification. *Comput. Statist. Data Anal.*, 66:55–69, 2013.

[24] James Hensman, Neil D Lawrence, and Magnus Rattray. Hierarchical bayesian modelling of gene expression time series across irregularly sampled replicates and clusters. *BMC Bioinformatics*, 14(1), August 2013.

[25] Bo Wang and Jian Qing Shi. Generalized Gaussian process regression model for non-Gaussian functional data. *J. Amer. Statist. Assoc.*, 109(507):1123–1133, 2014.

[26] Tarmo Äijö, Vincent Butty, Zhi Chen, et al. Methods for time series analysis of rna-seq data with application to human th17 cell differentiation. *Bioinformatics*, 30(12):i113–i120, 06 2014.

[27] Blake MacDonald, Pritam Ranjan, and Hugh Chipman. Gpfit: An r package for fitting a gaussian process model to deterministic simulator outputs. *Journal of Statistical Software*, 64(12):123, 2015.

[28] Abhirup Datta, Sudipto Banerjee, Andrew O. Finley, and Alan E. Gelfand. Hierarchical nearest-neighbor Gaussian process models for large geostatistical datasets. *J. Amer. Statist. Assoc.*, 111(514):800–812, 2016.

[29] Robert B. Gramacy. lagp: Large-scale spatial modeling via local approximate gaussian processes in r. *Journal of Statistical Software*, 72(1):146, 2016.

[30] Alexis Boukouvalas, James Hensman, and Magnus Rattray. Bgp: identifying gene-specific branching dynamics from single-cell data with a branching gaussian process. *Genome Biology*, 19(1), May 2018.

[31] Sumon Ahmed, Magnus Rattray, and Alexis Boukouvalas. Grandprix: scaling up the bayesian gplvm for single-cell data. *Bioinformatics*, 35(1):47–54, 07 2018.

[32] Lizhen Lin, Niu Mu, Pokman Cheung, and David Dunson. Extrinsic Gaussian processes for regression and classification on manifolds. *Bayesian Anal.*, 14(3):907–926, 2019.

[33] Atefeh Daemi, Hariprasad Kodamana, and Biao Huang. Gaussian process modelling with gaussian mixture likelihood. *Journal of Process Control*, 81:209–220, 2019.

[34] Lu Cheng, Siddharth Ramchandran, Tommi Vatanen, et al. An additive Gaussian process regression model for interpretable non-parametric analysis of longitudinal data. *Nature Communications*, 10(1), April 2019.

[35] Mark van der Wilk, Vincent Dutordoir, ST John, et al. A framework for interdomain and multioutput gaussian processes, 2020.

[36] Jiaxin Shi, Michalis Titsias, and Andriy Mnih. Sparse orthogonal variational inference for gaussian processes. In Silvia Chiappa and Roberto Calandra, editors, *Proceedings of the Twenty Third International Conference on Artificial Intelligence and Statistics*, volume 108 of *Proceedings of Machine Learning Research*, pages 1932–1942. PMLR, 2020.

[37] Nuha BinTayyash, Sokratia Georgaka, S T John, et al. Non-parametric modelling of temporal and spatial counts data from rna-seq experiments. *Bioinformatics*, 37(21):37883795, July 2021.

[38] Juho Timonen, Henrik Mannerström, Aki Vehtari, and Harri Lähdesmäki. lgpr: an interpretable non-parametric method for inferring covariate effects from longitudinal data. *Bioinformatics*, 37(13):1860–1867, 01 2021.

[39] Dimitrios V Vavoulis, Anthony Cutts, Jenny C Taylor, and Anna Schuh. A statistical approach for tracking clonal dynamics in cancer using longitudinal next-generation sequencing data. *Bioinformatics*, 37(2):147–154, 07 2020.

[40] Michele Vantini, Henrik Mannerström, Sini Rautio, et al. Pairgp: Gaussian process modeling of longitudinal data from paired multi-condition studies. *Computers in Biology and Medicine*, 143:105268, 2022.

[41] Allen Ross, Ali Rahnavard, and Jason Lloyd-Price. Representation learning with Gaussian processes in sparse irregularly sampled longitudinal metabolomics, 2022. the American Statistical Association Joint Statistical Meetings, Washington, DC, USA.

[42] J. T. Gene Hwang and Peng Liu. Optimal tests shrinking both means and variances applicable to microarray data analysis. *Stat. Appl. Genet. Mol. Biol.*, 9:Art. 36, 35, 2010.

[43] Guillermo Marshall and Anna E. Barón. Linear discriminant models for unbalanced longitudinal data. *Statistics in Medicine*, 19(15):1969–1981, 2000.

[44] Trevor Hastie, Robert Tibshirani, and Jerome Friedman. *The elements of statistical learning.* Springer Series in Statistics. Springer, New York, second edition, 2009. Data mining, inference, and prediction.

[45] Guillermo Marshall, Rolando De la Cruz-Mesía, Fernando A. Quintana, and Anna E. Barón. Discriminant analysis for longitudinal data with multiple continuous responses and possibly missing data. *Biometrics*, 65(1):69–80, 2009.

[46] Rolando. de la Cruz-Mesía and Fernando A. Quintana. A model-based approach to Bayesian classification with applications to predicting pregnancy outcomes from longitudinal β-hCG profiles. *Biostatistics*, 8(2):228–238, 04 2007.

[47] Luis Villarroel, Guillermo Marshall, and Anna E. Barón. Cluster analysis using multivariate mixed effects models. *Stat. Med.*, 28(20):2552–2565, 2009.

[48] Li-Xuan Qin and Steven G. Self. The clustering of regression models method with applications in gene expression data. *Biometrics*, 62(2):526–533, 2006.

[49] Christian P. Robert. *The Bayesian choice.* Springer Texts in Statistics. Springer, New York, second edition, 2007. From decision-theoretic foundations to computational implementation.

[50] David M. Blei, Alp Kucukelbir, and Jon D. McAuliffe. Variational inference: a review for statisticians. *J. Amer. Statist. Assoc.*, 112(518):859–877, 2017.

[51] Zhen Chen and David B. Dunson. Random effects selection in linear mixed models. *Biometrics*, 59(4):762–769, 2003.

[52] Paul-Christian Bürkner. Bayesian item response modeling in R with brms and Stan. *Journal of Statistical Software*, 100(5):1–54, 2021.

[53] Yeojin Chung, Sophia Rabe-Hesketh, Vincent Dorie, et al. A nondegenerate penalized likelihood estimator for variance parameters in multilevel models. *Psychometrika*, 78(4):685–709, 2013.

[54] Dimitris Rizopoulos. The r package jmbayes for fitting joint models for longitudinal and time-to-event data using mcmc. *Journal of Statistical Software*, 72(7):146, 2016.

[55] Dimitris Rizopoulos. Jm: An r package for the joint modelling of longitudinal and time-to-event data. *Journal of Statistical Software*, 35(9):133, 2010.

[56] Huang Xu, Xiang Li, Yaning Yang, et al. High-throughput and efficient multilocus genome-wide association study on longitudinal outcomes. *Bioinformatics*, 36(10):3004–3010, February 2020.

[57] Oludare Ariyo, Emmanuel Lesaffre, Geert Verbeke, and Adrian Quintero. Bayesian model selection for longitudinal count data. *Sankhya B*, 84(2):516–547, November 2021.

[58] Tanner Sorensen, Sven Hohenstein, and Shravan Vasishth. Bayesian linear mixed models using stan: A tutorial for psychologists, linguists, and cognitive scientists. *The Quantitative Methods for Psychology*, 12(3):175–200, October 2016.

[59] Nicholas Seedorff, Grant Brown, Breanna Scorza, and Christine A. Petersen. Joint bayesian longitudinal models for mixed outcome types and associated model selection techniques. *Computational Statistics*, September 2022.

[60] Michael I Love, Wolfgang Huber, and Simon Anders. Moderated estimation of fold change and dispersion for RNA-seq data with DESeq2. *Genome Biology*, 15(12), December 2014.

[61] Jason C. H. Tsang, Joaquim S. L. Vong, Lu Ji, et al. Integrative single-cell and cell-free plasma rna transcriptomics elucidates placental cellular dynamics. *Proceedings of the National Academy of Sciences*, 114(37), August 2017.

[62] Erika Kelmer Sacramento, Joanna M Kirkpatrick, Mariateresa Mazzetto, et al. Reduced proteasome activity in the aging brain results in ribosome stoichiometry loss and aggregation. *Molecular Systems Biology*, 16(6), June 2020.

[63] A. A. Tsiatis, Victor DeGruttola, and M. S. Wulfsohn. Modeling the relationship of survival to longitudinal data measured with error. Applications to survival and CD4 counts in patients with AIDS. *Journal of the American Statistical Association*, 90(429):2737, March 1995.

[64] Yan Wang and Jeremy M. G. Taylor. Jointly modeling longitudinal and event time data with application to acquired immunodeficiency syndrome. *J. Amer. Statist. Assoc.*, 96(455):895–905, 2001.

[65] Menggang Yu, Ngayee J. Law, Jeremy M. G. Taylor, and Howard M. Sandler. Joint longitudinal-survival-cure models and their application to prostate cancer. *Statist. Sinica*, 14(3):835–862, 2004.

[66] Michael S. Wulfsohn and Anastasios A. Tsiatis. A joint model for survival and longitudinal data measured with error. *Biometrics*, 53(1):330–339, 1997.

[67] Cong Xu, Pantelis Z. Hadjipantelis, and Jane-Ling Wang. Semi-parametric joint modeling of survival and longitudinal data: The r package jsm. *Journal of Statistical Software*, 93(2):129, 2020.

[68] S. M. Hammer, F. Vaida, K. K. Bennett, et al. Dual vs single protease inhibitor therapy following antiretroviral treatment failure: A randomized trial. *JAMA*, 288(2):169–180, 07 2002.

[69] Pulak Ghosh, Kaushik Ghosh, and Ram C. Tiwari. Joint modeling of longitudinal data and informative dropout time in the presence of multiple changepoints. *Stat. Med.*, 30(6):611–626, 2011.
